# Supplementary material for: The impact of conducting preclinical systematic reviews on researchers and their research: A mixed method case study
Source: PLoS One. 2021 Dec 13;16(12):e0260619. doi: 10.1371/journal.pone.0260619 (PMC8668092; doi:10.1371/journal.pone.0260619)
Supplement: S1 Appendix — (PDF) [file pone.0260619.s001.pdf]

S1 Appendix. The Research Impact Framework.

Table 1: Research Impact Framework – first main area of impact: research-related impacts (modified from Kuruvilla et al., 2007).

Research-related impacts	Definition of impacts
Type of problem/knowledge	-Provide data about a problem or phenomenon<br>- Evidence of effectiveness of interventions<br>- New research topics in a field<br>-Addressing research gaps and testing new hypotheses<br>-Create new ethical debates and guidelines<br>- Research topics of public, governmental, or media interest<br>- Making definitions and concepts<br>- Rationale for action/possible solutions<br>- Information on how to implement solutions
Research methods	-Replication of a study<br>-Application of established methods<br>-Further development/extension of methods<br>-Innovation e.g., animal-free alternatives<br>-Synthesis and methods development
Publications and papers	-Publications in scientific journals (and impact factors)<br>-Technical reports, project reports, position statements<br>-Citations of research publications
Products, patents and translatability potential	-Products and processes e.g., new product, improvement of a product, process innovation, opening new markets.<br>- Patents of research information or products and citations of patents<br>-Commercial development e.g., scientifically developed products, commercial licenses, spin-off companies<br>- Translatability potential: from basic science to clinical application, or technological outcomes and opportunities, e.g., publications in clinically oriented journal, influence on clinical trials, patent application and licenses
Research networks	Collaboration within and outside academia e.g., with other researchers, companies, funders, patients, policy-makers
Leadership and awards	-Role in setting the agenda or standards<br>-Leadership in coordinating and managing research projects and multi-institutional research collaborations<br>-Public recognition e.g., prestigious fellowship, named lecture, keynote speakers, membership of scientific society<br>- Membership of regional, national or international research bodies, review boards and funding bodies<br>- Editorship of journals or membership on journal editorial boards and advisory committees
Research management	-Expanding health research system linkages<br>-Changing research priority setting, investment strategies, resource allocation and accounting processes<br>- Developing capacities to conduct research and providing opportunities for training and development of researchers<br>- Changing the research environment e.g., working conditions, incentives, job retention rates<br>-Influencing health research system performance
Communication	-Dissemination of findings and knowledge, via all means and media
