# Supplementary material for: The impact of conducting preclinical systematic reviews on researchers and their research: A mixed method case study
Source: PLoS One. 2021 Dec 13;16(12):e0260619. doi: 10.1371/journal.pone.0260619 (PMC8668092; doi:10.1371/journal.pone.0260619)
Supplement: S2 Appendix — (PDF) [file pone.0260619.s002.pdf]

S2 Appendix. The Behaviour Change Wheel.

This framework is based upon a behavioural theory called "the COM-B system". This theory relies on the belief that if C (Capability), O (Opportunity) and M (Motivation) are combined for an individual, it will be possible to change behaviour, and that this change in behaviour will later influence their capability, opportunity, and motivation (Michie et al., 2011).

Capability is defined here as the possibility of engaging in a specific activity, including having the correct knowledge and skills. For example, a person would not be capable of joining a surfing class if they did not know how to swim, nor could a student start Master classes in theology without understanding what they entail.

Opportunity is defined as all external factors that may prompt or trigger behaviour, for instance unexpectedly meeting someone who is involved in charity events and starting to volunteer as a result.

Motivation is defined as all brain processes directing behaviour, such as habitual processes, emotional responses and analytical decision-making, for example being emotionally affected by constructive criticism and wanting to improve how to receive feedback as a result.

The framework, developed by Michie et al., (2011), emerged from a SR on behavioural change frameworks and is represented as a wheel with three distinct levels (Figure 2):

1) The required components for changing behaviour, as explained by the COM-B system

2) Interventions, i.e. activities aimed at changing behaviour

3) Policies, i.e. actions supporting or enabling the intervention and held (in part) by responsible authorities

In practice, there may be overlap between several of the given examples in the second level of the framework, i.e. between the different contexts of intervention. In the case of our intervention, the MKMD program can be defined as Imparting skills education, training and enablement.

- Education: "Increasing knowledge or understanding", via workshops, by improving awareness of and knowledge about preclinical SRs and their processes.

- Training: "Imparting skills", via workshops and coaching, by knowledge transfer on conducting preclinical SRs and doing so to a high standard.

- Enablement: "Increasing means/reducing barriers to increase capability or opportunity". This is accomplished via coaching and financial support, permitting researchers to perform their preclinical SRs (i.e. reducing the financial barriers and time constraints) and providing them with support (i.e. reducing the barrier of lack of experience).

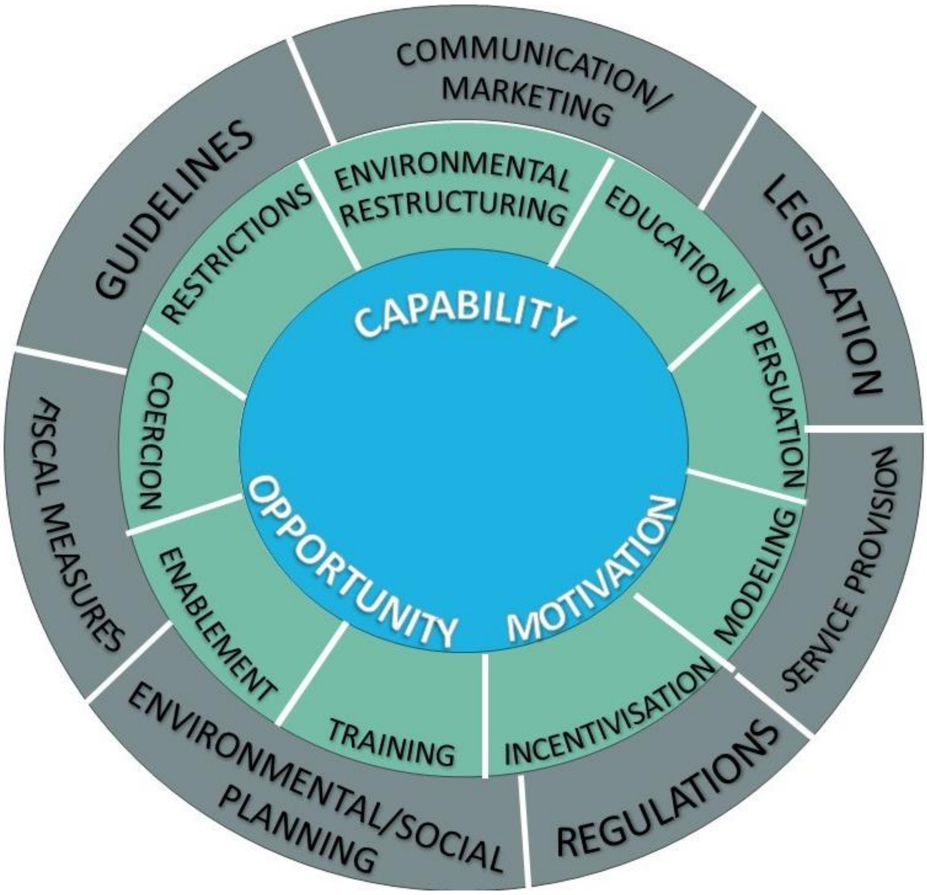

Fig 2. The Behaviour Change Wheel (modified from Michie et al., 2011).
The inner-circle represents the three requirements of behavioural change, the green circle the different contexts in which interventions take place, and the grey circle the different types of policies that can support or enable the interventions
