# Supplementary material for: The impact of conducting preclinical systematic reviews on researchers and their research: A mixed method case study
Source: PLoS One. 2021 Dec 13;16(12):e0260619. doi: 10.1371/journal.pone.0260619 (PMC8668092; doi:10.1371/journal.pone.0260619)
Supplement: S4 Appendix — (PDF) [file pone.0260619.s004.pdf]

S4 Appendix. Consolidated criteria for reporting qualitative studies (COREQ): 32-item checklist.

Developed from:

Tong A, Sainsbury P, Craig J. Consolidated criteria for reporting qualitative research (COREQ): a 32-item checklist for interviews and focus groups. *International Journal for Quality in Health Care*. 2007. Volume 19, Number 6: pp. 349 – 357

No. Item	Guide questions/description	Reported on Page #
**Domain 1: Research team and reflexivity**		
Personal Characteristics		
1. Inter viewer/facilitator	Which author/s conducted the interview or focus group?	P7, lines 137
2. Credentials	What were the researcher’s credentials? E.g. PhD, MD	P7, lines 138-139; lines 143-146
3. Occupation	What was their occupation at the time of the study?	P7 lines 137-146
4. Gender	Was the researcher male or female?	Refers to the pronouns “she” lines 138
5. Experience and training	What experience or training did the researcher have?	P7 lines 137-146
Relationship with participants		
6. Relationship established	Was a relationship established prior to study commencement?	No, P7 lines 139-141
7. Participant knowledge of the interviewer	What did the participants know about the researcher? e.g. personal goals, reasons for doing the research	P7, lines 141-142
8. Interviewer characteristics	What characteristics were reported about the interviewer/facilitator? e.g. Bias, assumptions, reasons and interests in the research topic	P7, lines 143
**Domain 2: study design**		
Theoretical framework		
9. Methodological orientation and Theory	What methodological orientation was stated to underpin the study? e.g. grounded theory, discourse analysis, ethnography, phenomenology, content analysis	P5
Participant selection		
10. Sampling	How were participants selected? e.g. purposive, convenience, consecutive, snowball	P8, lines 154-158
11. Method of approach	How were participants approached? e.g. face-to-face, telephone, mail, email	P8, lines 160-166
12. Sample size	How many participants were in the study?	In results,

		P11 lines 239-249, p12 lines 256-263 Fig2
13. Non-participation	How many people refused to participate or dropped out? Reasons?	P11 lines 239-247, Fig2
Setting		
14. Setting of data collection	Where was the data collected? e.g. home, clinic, workplace	P9, lines 177-178, lines 197-198
15. Presence of non-participants	Was anyone else present besides the participants and researchers?	P9, lines 198
16. Description of sample	What are the important characteristics of the sample? e.g. demographic data, date	P8, lines 155-158
Data collection		
17. Interview guide	Were questions, prompts, guides provided by the authors? Was it pilot tested?	P9-10 lines 198-202, P11 lines 231-233 + acknowledgements
18. Repeat interviews	Were repeat interviews carried out? If yes, how many?	N/A
19. Audio/visual recording	Did the research use audio or visual recording to collect the data?	P10, lines 210-211
20. Field notes	Were field notes made during and/or after the interview or focus group?	P10, lines 201-202
21. Duration	What was the duration of the inter views or focus group?	P9, lines 197
22. Data saturation	Was data saturation discussed?	P8, lines 165-166
23. Transcripts returned	Were transcripts returned to participants for comment and/or correction?	P10, lines 212-213
**Domain 3: analysis and findings**		
Data analysis		
24. Number of data coders	How many data coders coded the data?	P10, lines 214-216.
25. Description of the coding tree	Did authors provide a description of the coding tree?	S9 Appendix
26. Derivation of themes	Were themes identified in advance or derived from the data?	P10, lines 214-216.
27. Software	What software, if applicable, was used to manage the data?	Atlas.ti, lines 2015
28. Participant checking	Did participants provide feedback on the findings?	P10, lines 217-218
Reporting		
29. Quotations presented	Were participant quotations presented to illustrate the themes/findings? Was each quotation identified? e.g. participant number	Results; yes, p13-14 (lines 294-302); 17-23
30. Data and findings consistent	Was there consistency between the data presented and the findings?	Yes, Discussion p23-24
31. Clarity of major themes	Were major themes clearly presented in the findings?	Yes, Fig5
32. Clarity of minor themes	Is there a description of diverse cases	Yes, p16-23

	or discussion of minor themes?	
