# Supplementary material for: The impact of conducting preclinical systematic reviews on researchers and their research: A mixed method case study
Source: PLoS One. 2021 Dec 13;16(12):e0260619. doi: 10.1371/journal.pone.0260619 (PMC8668092; doi:10.1371/journal.pone.0260619)
Supplement: S5 Appendix — (PDF) [file pone.0260619.s005.pdf]

S5 Appendix. Invitation to complete the online questionnaire and reminder.

Dear Sir/Madam XX,

My name is Julia Menon, and I am a research assistant. I'm currently performing a case study, on behalf of ZonMw, regarding their funding program "Meer Kennis met Minder Dieren (MKMD)".

ZonMw aims to evaluate the impact of performing synthesis of evidence / systematic reviews of animal studies (and preclinical studies) on researchers and their research.

In this context, we are approaching all researchers who performed or are performing systematic reviews with ZonMw funding and/or coaching.

Therefore, we kindly invite you to fill out an online questionnaire. We would be very grateful if you could take the time to participate.

The questionnaire should take about **15 min** only and can be found at the following link: https://www.questionpro.com/t/AQwH0ZiBJV
Please, fill the questionnaire online **before the 23rd of August**.

All your information will be kept anonymous and confidential. The only purpose of this analysis will be to produce an internal report for ZonMw.

If you need any extra information, feel free to contact me at this e-mail or at the following email:
Erica van Oort (ZonMw) at mkmd@zonmw.nl

Thanking you in advance,

Yours Sincerely,

Julia Menon

Reminder

Dear Sir/Madam XX

You were sent an invitation 2 weeks ago to participate in a questionnaire, with the aim to evaluate the impact of ZonMw's program on performing synthesis of evidence / systematic reviews on researchers and their research. We understand that you may be busy or unavailable due to summer plans, therefore this kind reminder. We sincerely hope you can participate during the summer season.

If you need any extra information, feel free to contact me at this e-mail or at the following emails:
Erica van Oort at mkmd@zonmw.nl

Thank you in advance for your time,

Yours Sincerely,

Julia Menon
