# Supplementary material for: The impact of conducting preclinical systematic reviews on researchers and their research: A mixed method case study
Source: PLoS One. 2021 Dec 13;16(12):e0260619. doi: 10.1371/journal.pone.0260619 (PMC8668092; doi:10.1371/journal.pone.0260619)
Supplement: S6 Appendix — (PDF) [file pone.0260619.s006.pdf]

S6 Appendix. Full questionnaire.

Dear researcher who received funding from ZonMw to conduct a systematic review,

You are invited to participate in our questionnaire, which aims to gather information for a case study report on the ZonMw funding program, “Meer Kennis met Minder Dieren (MKMD)“. It should take about 15 minutes to complete.

The main topic of the study is to evaluate the **impacts of performing a systematic review of preclinical studies**. It aims to assess both **impacts on research** (e.g. methods and models used after performing a systematic review) and **impacts on the researcher** (e.g. point of view, behaviour in research such as reporting, writing, research appraisal after completing the review).
Therefore, you will be asked several questions about your opinions and behaviour after performing your systematic review, such as designing and planning experiments, writing papers, appraising research and your personal experiences with performing a systematic review.

Do not worry if you have not finished your systematic review as yet or if it stopped ! Your input is still highly valuable feedback.

Your participation in this study is completely voluntary. If you feel uncomfortable answering any questions, you can withdraw from the survey at any point. Additionally, all your information will be kept anonymous and confidential, as this research follows and complies with the data protection act of 2018 and is solely for internal use within ZonMw.

We kindly thank you for your participation and your time!
Please start with the questionnaire now by clicking on the “Next” button below.

Introductory question

Have you completed a systematic review of preclinical studies? (*)

Yes ☐ No ☐

Have you performed primary animal experiments after conducting your systematic review?

Yes ☐ No ☐

--Page Break--

Designing and planning experiments

The following section focuses on your research projects during or after completing the preclinical systematic review. Questions are centred around the topics you addressed, the way you planned your experiments and methods and models you chose.

Please choose the answer that best fits the following statements: (*)

Conducting a systematic review had an impact on…	*Completely Disagree*	*Mostly Disagree*	*Slightly Disagree*	*Neither disagree nor agree*	*Slightly Agree*	*Mostly Agree*	*Completely Agree*
The topics I addressed in (future) research	☐	☐	☐	☐	☐	☐	☐
The use of a planning guideline, e.g. PREPARE, for my following research	☐	☐	☐	☐	☐	☐	☐
The time allocated for planning my following research	☐	☐	☐	☐	☐	☐	☐
The procedure I used for planning my following research	☐	☐	☐	☐	☐	☐	☐
The animal modell chose inmy following research	☐	☐	☐	☐	☐	☐	☐
The intervention I chose in my following research	☐	☐	☐	☐	☐	☐	☐
The use of blinding inmy following experiments	☐	☐	☐	☐	☐	☐	☐
The use of randomization in my following experiments	☐	☐	☐	☐	☐	☐	☐
The use of power calculation in my following experiments	☐	☐	☐	☐	☐	☐	☐
Other experimental design aspects you chose inmy following experiments	☐	☐	☐	☐	☐	☐	☐
Avoiding duplication of (part of) the investigation	☐	☐	☐	☐	☐	☐	☐

If applicable, for the questions you answered with “mostly agree” or completely agree” could you briefly explain your answers?

--Page Break--

Writing manuscripts

The following section will focus on the primary study papers you wrote and their content after completing your preclinical systematic review.

Please choose the answer that best fits the following statements: (*)

When writing subsequent papers, conducting a systematic review had an impact on…	*Completely Disagree*	*Mostly Disagree*	*Slightly Disagree*	*Neither disagree nor agree*	*Slightly Agree*	*Mostly Agree*	*Completely Agree*
My use of reporting guideline, e.g. ARRIVE	☐	☐	☐	☐	☐	☐	☐

Reporting an ethical statement	☐	☐	☐	☐	☐	☐	☐
Reporting a justification for the chosen animal model							
Reporting my animal model provider	☐	☐	☐	☐	☐	☐	☐
Reporting housing conditions	☐	☐	☐	☐	☐	☐	☐
Reporting my animal model characteristics (e.g. species, sex, weight)	☐	☐	☐	☐	☐	☐	☐
Reporting of randomization	☐	☐	☐	☐	☐	☐	☐
Reporting of blinding	☐	☐	☐	☐	☐	☐	☐
Reporting of a power calculation/sample size calculation	☐	☐	☐	☐	☐	☐	☐
Publishing in an open access journal	☐	☐	☐	☐	☐	☐	☐
If applicable, publishing negative data	☐	☐	☐	☐	☐	☐	☐

--Page Break--

Have you performed preclinical research with alternatives to animal models after conducting your systematic review?

Yes ☐ No ☐

If "yes": did your systematic review influence your choice to use an alternative model?

Yes ☐ No ☐

If "yes": please explain **how** your systematic review influenced your choice of model?

Have you performed clinical research after conducting your systematic review?

Yes ☐ No ☐

If "yes" how did the SR impact your clinical research?

Appraising research

The following section focuses on the way you appraise research after performing a preclinical systematic review.

Please choose the answer that best fits the following statements: (*)

When appraising research papers, conducting a systematic review had an impact on…	*Completely Disagree*	*Mostly Disagree*	*Slightly Disagree*	*Neither disagree nor agree*	*Slightly Agree*	*Mostly Agree*	*Completely Agree*
Your critical sense when reading research papers	☐	☐	☐	☐	☐	☐	☐
Your critical sense towards peers' research	☐	☐	☐	☐	☐	☐	☐
Your critical sense toward your own previous research	☐	☐	☐	☐	☐	☐	☐
The way you appraise the overall research quality in your field	☐	☐	☐	☐	☐	☐	☐

---Page Break---

Your skills after conducting the preclinical systematic review

The following section focuses on skills you may have acquired by conducting your preclinical systematic review.

Did you **learn** new research skills by conducting the systematic review? (*)

Yes ☐ No ☐ No opinion ☐

If yes, what new research skills did you learn?

Did you **improve** your existing research skills by conducting the systematic review? (*)

Yes ☐ No☐ No opinion☐

If yes: what research skills did you improve?

Did you **learn** new skills beyond research skills by conducting the systematic review? (*)

Yes ☐ No ☐ No opinion ☐

If yes: what new skills did you learn?

Did you **improve** existing skills beyond research skills by conducting the systematic review? (*)

Yes ☐ No ☐ No opinion☐

If yes: what skills did you improve?

Your experience with conducting a preclinical systematic review

The following section focuses on your personal experience of performing a preclinical systematic review. It addresses questions about your experience with the coaching, your personal opinion and future perspectives.

Conducting and publishing the review

Please choose the answer that best fits the following questions:

Did completing the review match your expectations in term of duration? (*)

Yes ☐ No, it was longer ☐ No, it was shorter ☐

If "No": Can you please state how much (how many weeks) longer or shorter?

Which stages of the systematic review went slower or faster than expected? (multiple answers can be picked)

☐ Writing protocol

☐ Searching for all the evidence

☐ Study selection / screening

☐ Data extraction study characteristics

☐ Risk of bias assessment/study quality assessment

☐ Data extraction of outcome data

☐ Data analysis/synthesis

☐ Writing the manuscript

☐ Publishing the manuscript

Please tell us briefly what happened:

What was your experience with publishing your systematic review? (response of both journals/editors and reviewers)

Opinions and future perspectives

Would you recommend conducting a preclinical systematic review to your colleagues/peers? (*)

Yes ☐ No ☐ Maybe ☐ No opinion ☐

Is it likely that you will conduct another preclinical systematic review? (*)

Yes ☐ No ☐ Maybe ☐ I already conducted another review ☐ No opinion☐

If "yes": Could you please explain why?

If "no": Could you please explain why not?

If "maybe": Could you please explain why?

If "I already conducted another review":

How many preclinical reviews did you conduct? (please fill in the corresponding number)

If "I already conducted another review":

Did you complete this/these systematic review(s)?

☐ Yes, I did

☐ No, it is a current project

☐ No, the project stopped (please specify why) _________________

☐ Other (please specify) _____________

Which factors would encourage you to conduct another preclinical systematic review?

What was your experience with the coaching?

Would you like to receive coaching again? (*)

Yes ☐ No ☐ Only for some stages of the systematic review ☐ No opinion ☐

If "only for some stages of the systematic review": for which stages would you like support?

☐ Writing protocol

☐ Searching for all the evidence

☐ Study selection / screening

☐ Data extraction of study characteristics

☐ Risk of bias assessment/study quality assessment

☐ Data extraction of outcome data

☐ Data analysis/synthesis

☐ Writing the manuscript

☐ Publishing the manuscript

☐ Other (please specify) ____________

For further analysis of this topic, we would like to perform interviews (± 1 hour) with some of the participants of this questionnaire.

Would you in principle be willing to take part in a semi-structure interview concerning this topic? If yes, you will be sent all of the required information in the near future.

Yes ☐ No☐

Please give us your e-mail so we can contact you regarding the interview.

---Page Break---

Thank you for completing this questionnaire

We are very grateful for your contribution and your time

Branching: if they answered no to the introductory question

Your ZonMw funded preclinical systematic review is ….

ongoing ☐ stopped ☐

If "ongoing":

Please provide information regarding the stage of your systematic review (*)

Stages	*Stage started*	*Stage finalized*
Writing protocol	☐	☐
Searching for all the evidences	☐	☐
Study selection / screening	☐	☐
Data extraction of study characteristics	☐	☐

Risk of bias assessment/study quality assessment	☐	☐
Data extraction of outcome data	☐	☐
Data analysis	☐	☐
Writing the manuscript	☐	☐

If “stopped”:

Could you please tell us why the systematic review stopped?

--Page Break---

Do you think that conducting this systematic review will have an impact on the way you design, conduct and/or report future experiments?

Yes ☐ No ☐ Maybe ☐ No opinion ☐

If “yes” or “maybe”: Please explain briefly how

Appraising research

The following section focuses on the way you appraise research now that you are accustomed to preclinical systematic review methods.

<u>Please choose the answer that best fits the following statements: </u>(*)

When appraising research papers, performing a systematic review had an impact on…	*Completely Disagree*	*Mostly Disagree*	*Slightly Disagree*	*Neither disagree nor agree*	*Slightly Agree*	*Mostly Agree*	*Completely Agree*
Your critical sense when reading research papers	☐	☐	☐	☐	☐	☐	☐
Your critical sense towards peers’ research	☐	☐	☐	☐	☐	☐	☐
Your critical sense toward your own research	☐	☐	☐	☐	☐	☐	☐
The way you appraise overall research quality in your field	☐	☐	☐	☐	☐	☐	☐

--Page Break---

Skills after performing the preclinical systematic review

Did you **learn** new <u>research skills</u> by conducting the systematic review? (*)

Yes ☐ No ☐ No opinion☐

If yes, what new research skills did you learn?

Did you **improve** your research skills by conducting the systematic review? (*)

Yes ☐ No☐ No opinion☐

If yes: what research skills did you improve?

Did you **learn** new skills beyond research skills by conducting the systematic review? (*)

Yes ☐ No ☐ No opinion ☐

If yes: what personal skills did you learn?

Did you **improve** your skills beyond research skills by conducting the systematic review? (*)

Yes ☐ No ☐ No opinion☐

If yes: what personal skills/soft skills did you improve?

Your experience with conducting a preclinical systematic review

The following section focuses on your personal experience of performing a systematic review. It addresses questions about your experience with the coaching, your personal opinion and future perspectives.

Conducting the systematic review

Please choose the answer that best fits the following questions:

So far, did the review match your expectation in term of duration? (*)

Yes ☐ No, it was longer ☐ No, it was shorter ☐

If "No": Which stages of the systematic review went slower or faster than expected? (multiple answers are possible/can be picked)

☐ Writing protocol

☐ Searching for all the evidence

☐ Study selection / screening

☐ Data extraction of study characteristics

☐ Risk of bias assessment/study quality assessment

☐ Data extraction of outcomes data

☐ Data analysis

☐ Writing the manuscript

Please tell us briefly what happened:

Opinions and future perspectives

Would you recommend conducting a preclinical systematic review to your colleagues/peers? (*)

Yes ☐ No ☐ Maybe ☐ No opinion ☐

Is it likely that you will conduct more preclinical systematic reviews in the future? (*)

Yes ☐ No ☐ Maybe ☐ No opinion ☐

If "yes": Could you please explain why?

If "no": Could you please explain why not?

If "maybe": Could you please explain why?

Which factors would encourage you to conduct another preclinical systematic review?

--Page Break--

Thank you for completing this questionnaire

We are very grateful for your contribution and your time
