# Supplementary material for: The impact of conducting preclinical systematic reviews on researchers and their research: A mixed method case study
Source: PLoS One. 2021 Dec 13;16(12):e0260619. doi: 10.1371/journal.pone.0260619 (PMC8668092; doi:10.1371/journal.pone.0260619)
Supplement: S7 Appendix — (PDF) [file pone.0260619.s007.pdf]

S7 Appendix. Informed consent form.

Informed Consent Form for Participants in the Qualitative Study

"Conducting preclinical systematic reviews – the impact on research and researchers "

Principal Investigator

Julia Menon on behalf of ZonMw; Erica van Oort, ZonMw representative

Telephone: +33 6 26 36 84 38

Julia.menon@radboudumc.nl

mkmd@zonmw.nl

This Informed Consent Form has two parts:

• Information Sheet (to share information about the study with you)

• Certificate of Consent (for signatures if you choose to participate)

You will be given a copy of the full informed consent form

Part I: Information Sheet

You are being asked to take part in a research study. Before you decide to participate in this study, it is important that you understand why the research is being done and what it will involve. Please read the following information carefully and ask the researcher if anything is not clear or if you need more information.

Introduction

I'm Julia Menon, research and teaching assistant. I'm currently performing a case study for ZonMw regarding their funding program "Meer kennis met minder dieren (MKMD)/More Knowledge with Fewer Animals".

Purpose of the Study

The ZonMw funding program "MKMD" has been running since 2012. Within the program the Module 'kennisinfrastructuur'/knowledgeinfrastructure is focused on education and coaching for preclinical systematic reviews and funded many projects in the Netherlands. ZonMw would like to evaluate the reach of their program, notably by assessing the usefulness of funding preclinical systematic reviews. Therefore, we aim to investigate the impact of conducting systematic reviews, both on research and the researchers.

Procedure

This research will involve your participation in a one-hour interview, where you are kindly asked to respond to several open-questions. During the interview, I (Julia Menon) will interview you

via teleconference, using *GoTo meetings,* and will record our exchange for further analysis. If you do not wish to answer any of the questions during the interview, you may say so, and we will move on to the next question. No one else but I will be present unless you would like someone else to be there. We can then invite them to the call.

The information recorded is confidential; it will be transcribed verbatim. Only the ZonMw representative for this project (dr. Erica van Oort) will have access to the recording and the information documented during your interview. Additionally, your information will be used for an internal ZonMw report only. The entire interview will be recorded, but no-one will be identified by name in the data analysis or in any other future steps of publication. The recording will be destroyed after 16 weeks.

Participant Selection

You are being invited to take part in this research because we feel that your experience with the preclinical systematic reviews would be highly valuable. In particular, to highlight how conducting a systematic review impacted your subsequent projects and your opinion on (the quality of published) animal research.

Voluntary Participation

Your participation in this study is voluntary. It is up to you to decide whether or not to take part in this study. If you choose to take part, you will be asked to sign this informed consent form. After you sign the consent form, you are still free to withdraw at any time and without giving a reason. Withdrawing from this study will have no bearing on your job or any work-related evaluations or report. If you withdraw from the study before data collection is completed, your data will be returned to you or destroyed.

Risks

The questions asked during the interview will be related to your experience with conducting your ZonMw funded/coached systematic review, and on the impacts it may have had on your subsequent research project and yourself. If you do not feel comfortable with this topic for personal, work-related, or other reasons, you may decline to answer any or all questions, and you may terminate your involvement at any time.

Benefits

There will be no direct benefit to you, but your participation is likely to help us identify the impacts systematic reviews may have and can support ZonMw to continue and/or reform the current grants.

Confidentiality

Confidentiality, privacy and anonymity is the main priority in our study. Every effort will be made by the researcher to preserve your confidentiality by the following:

- Assigning a code name to your collected information, as well as on all research notes and documents
- No mention of your profession, age, or any distinct characteristic that may be associated with your person.
- Interviews will be done and recorded on a trustworthy software.
- Digitally secure your data
- Access will be limited to the ZonMw representative (dr. Erica van Oort), and I (Julia Menon)
- Ensure destruction of the recording within 16 weeks after the original interview.

Participant data will be kept confidential except in cases where the researcher is legally obligated to report specific incidents. These incidents include, but may not be limited to, incidents of abuse and suicide risk.

Contact Information

If you have questions at any time about this study, you may contact the researcher whose contact information is provided on the first page.

Part II: Certificate of Consent

To be filled by the participant

I have read and I understand the provided information and have had the opportunity to ask questions.

I understand that my participation is voluntary and that I am free to withdraw at any time, without giving a reason and without cost.

I have had the opportunity to ask questions and any questions I had have been answered to my satisfaction.

I understand that I will be given a copy of this consent form.

I voluntarily agree to take part in this study.

Name of Participant ______________________________

Signature

Date ______________________________

To be filled by the investigator

I confirm that the participant was given an opportunity to ask questions about the study, and all the questions asked by the participant have been answered correctly and to the best of my ability. I confirm that the individual has not been coerced into giving consent, and the consent has been given freely and voluntarily.

Name of Investigator______________________________

Signature

Date ______________________________
