# Supplementary material for: The impact of conducting preclinical systematic reviews on researchers and their research: A mixed method case study
Source: PLoS One. 2021 Dec 13;16(12):e0260619. doi: 10.1371/journal.pone.0260619 (PMC8668092; doi:10.1371/journal.pone.0260619)
Supplement: S8 Appendix — (DOCX) [file pone.0260619.s008.docx]

**S8 Appendix. Interview guide.**

Interview Guide

Note for the researcher: green text in italics is not said out loud but only an indication. Bullet points under some questions are meant to assure that discussion does not become out of topics + give the possibility to ask open questions on the go

**Introduction**

Good morning/good afternoon

Thank you for giving me some of your time today. Before the interview, we will first read through the informed consent form together to provide you with a brief recap of the goal of our study and the setting of the interview. If you have any question, please do not hesitate.

Do you have any questions for the moment?

*Read the ICF*

Is the aim of the interview clear?

*Start the recording*

**Breaking of the ice and setting context**

1. To start, could you please briefly introduce yourself?

- Their background
- Their role in their institute

In the first part of the interview, I will ask you some questions regarding your experience with the systematic review, and then in a second part, we’ll discuss the impact of systematic reviews. Some of these questions you already answered in the questionnaires, but I would like to know your opinion further regarding these topics.

**Experience with the systematic review**

1. So, we’ll start with some questions about your systematic review:
   1. Could you please tell me about the topic of your systematic review and when you started this project?
   2. Why have you conducted this systematic review?

- What factors incited you to conduct this SR?

1. Now we’ll focus on more details with the content of your review, in particular on the results that you had.

As you know, when starting a systematic review, you need to write a precise protocol, which helps to lay out what will be done in a transparent way. However, it can be difficult to predict what you will find.

- 1. In your case, did your expectations align with your final results?
- *Number of studies lower/higher than expected:* Was the number of studies lower or higher than expected?
- *Studies available or unfindable full text:* Did you have any issues with collecting all studies full text?
- *Quality of studies:* What did you expect from the quality of studies?
- *Unexpected findings*: Did you encounter any unexpected findings?
  1. Was there sufficient evidence to draw conclusions?
  2. Was there enough quality to draw conclusions?

1. Now I would like to ask you more in-depth questions regarding your experience with doing a systematic review, in particular related to your skills and insights.

By doing this review, what insights did you gain?

- *Awareness:* How has it changed your awareness/experience in your field?
- *Critical sense*: Did it change your critical sense?
- *Skills:* Which of your skills did you gain or improve since you are familiar with the methods of a systemic review?

**Impacts – change in behaviour/attitude**

We’ll continue with the second part of the interview. We will focus on the insights you gained from this systematic review and how they impact your subsequent research projects, including the ones you’re doing right now. Could you please tell me what type of project your conducted beside or after your review? E.g. animal studies, clinical studies etc.

- 1. After your review or beside it, what type of research did you do?
     1. For instance, animal research, clinical research.

*If they use animal models:*

- 1. Since you have completed your systematic review, do you consider using alternatives to animal models?

If yes: Why? Are any alternatives available?

1. Has your opinion changed regarding the overall quality of animal research?
2. In what way did the insights that you got from the systematic review influence the way you conduct animal research?

- *Model/methods:* Did it impact the models and methods you chose? Could you please elaborate?
- *Critical planning:* did it impact the way you plan future experiments? How?
- *Reporting:* did impact the way you reported your model and methods? How?
- *Make it easily accessible:* did it influence you to make your next research accessible?
- *Avoid duplication:* did it enable you to avoid duplication?

1. As said earlier, results gained from a systematic review can be unpredictable and are prone to generate new hypotheses. In your case, did conducting this systematic review give directions to your future projects or inspire new projects?

- *Topic:* Did it change the topic of your research? E.g. you used to focus on protein A and now on protein B.
- *Models:* Did you base your research on other animal models? E.g. discover that animal A is better suited than previous model they were using
- *Further methods*: Did it trigger the development of further methods? E.g. realise that no induction of a model was done during the treatment and decided to write a protocol for it
- *Project outside animal*: Did it trigger the start of new projects outside of animal research? E.g. make a letter, a mobile app.

1. You were talking earlier about how much insights you gained by conducting this systematic review.
   1. Is it common to perform systematic reviews of animals in your field?
      1. How is it received by journals or reviewers?
   2. How valuable do you think systematic reviews of animals are in your field?

- Can an SR be of any benefit for translational purposes?
  1. In your opinion, what can be done to promote systematic reviews of animals in your field?

1. Do you have any ideas or comments you could add about this topic that we did not yet discuss?

**Conclusion**

Thank you for your participation and for allowing me to use some of your time. This information is very valuable to us. Would you like to be kept up to date with the results of the project?

*Stop recorder*
