# Supplementary material for: The impact of conducting preclinical systematic reviews on researchers and their research: A mixed method case study
Source: PLoS One. 2021 Dec 13;16(12):e0260619. doi: 10.1371/journal.pone.0260619 (PMC8668092; doi:10.1371/journal.pone.0260619)
Supplement: S9 Appendix — (PDF) [file pone.0260619.s009.pdf]

S9 Appendix. Code tree and harmonisation.

How impact occur: Trigger: workshops (education) + coaching and funding (enablement) → Participant perform SR → opinion change/get insight/realize things → implement change in their habits ➔ Impact (+ impact in their field/at a broader level)

Lab level / team level

- **Experience with the review**
  - Characteristics of their review/how it went (Black colour)
    - *SR Bigger than planned*
    - *Narrowed down SR*
    - *Change SR methodology*
    - *Collect FT*
    - *Correct for bias*
    - *Delay in SR*
    - *Unpredictability of SR*
    - *SR stages are time consuming*
    - *Having good knowledge of the field helps for SR*
    - *Like the transparency of SR*
    - Unexpected results
    - Positive feedback to SR promotion
  - Motivation/reason to do SR → start code by "reason to do SR:" (Pink colour)
    - *Reason to perform SR: boost in vitro model*
    - *Reason to perform SR: create an alternative model*
    - *Reason to perform SR: place negative data into perspective*
    - *Reason to perform SR: prepare primary study*
    - *Reason to perform SR: preclinical overview before clinical study*
    - *Reason to perform SR: understand preclinical heterogeneity*
    - *Reason to perform SR: got hyped by the workshop*
    - *Reason to perform SR: ideas from previous studies*
    - *Reason to perform SR: do mere science*
  - Draw conclusion
    - *Draw conclusion: bad quality*
    - *Draw conclusion: enough data*
    - *Lack of evidence for analysis*
  - Realisation of badly conducted/reported studies
    - *Bad reporting*
    - *Bad study design*
    - *Bad study design: underpowered studies*

- Increase/improve skills
  - *Skills: meta-analysis*
  - *Skills: become more critical*
  - *Skills: change in attitude, more optimistic*
  - *Skills: more aware on biases*
  - *Skills: pre-registration → i.e. protocol for SR*
  - *Skill: comprehensive search*
  - *Learning curve*
  - *Protocol are useful*

- Insight they gained
  - Code “insights”
    - *Insight: confirm theories*
    - *Insights: debunk theories*
    - *Insights: highlight heterogeneity/differences*
    - *Insights: highlight discrepancies*
    - *Insights: highlight that induced model do not match human situation*
    - *Insights: identify data gaps*
    - *Insights: increased awareness for their field*
    - *Insights: create new data without using new animals*
    - *Insights: didn’t realise how much time/effort for SR*
    - *Insights: training is needed to do SR*

- Realize things
  - *Realize impact on animals*
  - *Realize own past mistakes*
  - *“quality is worse than I thought”*
  - *SR can have mistake/restriction*
  - *SR are valuable / valuable overview*
    - *Insights: create new data without using new animals*
  -
  - *Valuable for translation*
  - *If SR give you answer, why perform animal research?*

→this change in perspective/opinion, thanks to the intervention results in the code below:

- **SR impact the way people**
  - Conduct (Blue colour)
    - Intervention
      - *SR change a “standard” method*
      - *SR helps to create new intervention methods*
    - Animals

            - *SR improves choice of animal model*
        - Design
            - *SR improve design*
    - Plan
        - Preparation before experiment:
            - *Perform SR before primary study*
                - ➔ *Use SR to ask relevant question*
            - *Use/perform SR to avoid duplication*
            - *Insights: create new data without using new animals*

        - Choose model:
            - *SR highlights unethicality of some models*
            - *Alternatives to animal models*
            - *Alternatives not available*

        - Topic addressed
            - SR impacts future topics/investigations
            - SR leads to new "targets"

        - Design
            - *SR improve design*

    - Report
        - SR improved: reporting of experiment
    - Appraise
        - Opinion about quality
            - *"quality was worse than I thought"*
            - *Quality is better than they thought*
        - The way they review research
            - *SR teach reviewers how to improve*
        - May use SR in future to address quality
            - SR to address quality

- **Make them switch field**
    - *Switching fields after SR*

- **SR give them new ideas for**
    - Other SRs
        - *SR led to new SRs*
    - Methodological papers/statistical papers
        - *SR lead to methodology development*
    - Translational ideas
        - *careful when using SR for translational purposes*
        - *Translational SR includes animals and humans*

- **Advocate change in their team**
  - *Professor do not realize the value of SR*
  - *Teaching SR to others*
  - *Young researchers are the future of SRs*

- **Collaboration**
  - *Create contact via SRs* (1 example of contact via publication / 1 example of contact via presentation at conference)

Field level

- **Observation of their field:** (Orange colour)
  - *Transparency/quality become better* → with time
  - Why people don't adopt SR/improve experiments
    - *Lab have convention on models/methods*
    - *Lie on reporting*
    - *People don't adapt new ways due to pressure*
    - *People do animal experiment by convenience*
    - *People don't know how to report without proper guidance*
    - *People don't realize the value of SR*
  - *People do SR only for personal gain*
  - *People do their best, their don't underreport for fun*

- **Place of SRs in their field**
  - Common/uncommon?
    - *SR of animals not common*
    - *SR are increasing in their field*
    - *Uptake is slow for SR*
    - *Don't overdo SR of animals*
    - *People see the value of SR*
  - Valuable for translation or change
    - *SR results do not impact the field*
      - *See quote ZonMw23 : SR change a "standard" method*
    - *Use SR for translational purposes*

  - Acceptance by journals and editors

    - *Editor not knowledgeable about SR*
    - *Return by reviewers: good*
    - *Return by reviewers: made SR more robust*
    - *People don't realize the value of SR*
    - *Journals don't accept SR*

        - *Problem to publish*
        - *Transparency comes from 2 ways*

- **Dissemination (of SR – the concept /of the results of their SR)**
    - Publish in journals (Red colour)
        - *Push SR results into "research agenda"*
            - *Get ethical committees involved*

    - Awareness for researchers
        - Code: "ways to promote:"
            - Ways to promote: lectures
                - Ways to promote: article 9
            - Ways to promote: workshops
            - Ways to promote: website
            - Ways to promote: social media
            - Ways to promote: conference
            - Ways to promote: advise peers to do SR
            - Ways to promote: enable SR via funding
            - Ways to promote: show SR value
            - Ways to promote: via the MKMD scheme

    - Target population
        - Code "Upgrade target population grant"
            - Upgrade target population grant: PhD student
            - Upgrade target population grant: medical student/graduate student
            - Upgrade target population: professor, PI
            - Upgrade target population grant: organization

- **Advocate change in their field** (Yellow colour)
    - Make opinion paper or letter→ or editorial
        - *Publish opinion or letter to change views of SR in the field*
    - Promote alternatives
        - *Promote alternatives (funders)*
        - *Promote alternatives (journals)*
        - *Promote alternatives (researchers)*
        - *Promote meta-research to reduce animal use*
    - *Promote pre-registration*
    - *Promote use of SR results further* → by making apps, proposing "live SR", making all SR data usable
    - *Responsibility to do SR if they can*

Science community level

- Bring research forward: (not CODE: observation)
    - By sending it to the right people → regulation, change in status quo (e.g. SR mandatory for the CDD application)

- Dissemination (Green colour)
    - *University need to open upgrade and open to innovation*
    - *Set SRs as a requirement before animal studies*
    - *Get ethical committees involved*
    - *Education could increase transparency*

Refinement of the grant/comment about the grant

- Coaching (Purple color)
    - Coaching: Praise coaching
    - Coaching/workshops are limited in their collaboration
    - Allocate hours of coaching better
    - Coacher less overloaded
    - Coaching should not be a requirement
    - Prioritize coaching for newbies to SR
- Grant scheme
    - Different approach with the funding scheme
    - Expend MKMD to method development
    - Expend MKMD to other type of review
    - Praise MKMD
    - Give funding to SR
    - Increase time of the MKMD program

- Things ZonMw could do to enforce SR
    - ZonMw enforce SR for people who ask for funding for animal studies
    - ZonMw organise research collaboration
    - ZonMw looks critically of the finding of SRs to influence future preclinical studies

Other

(Brown colour)

- In vitro lacking behind
- Don’t like pre-registration
