# Supplementary figures and images for: The impact of conducting preclinical systematic reviews on researchers and their research: A mixed method case study
Source: PLoS One. 2021 Dec 13;16(12):e0260619. doi: 10.1371/journal.pone.0260619 (PMC8668092; doi:10.1371/journal.pone.0260619)

**S10 Appendix. Organisation of the questionnaire and number of respondents per questions.**

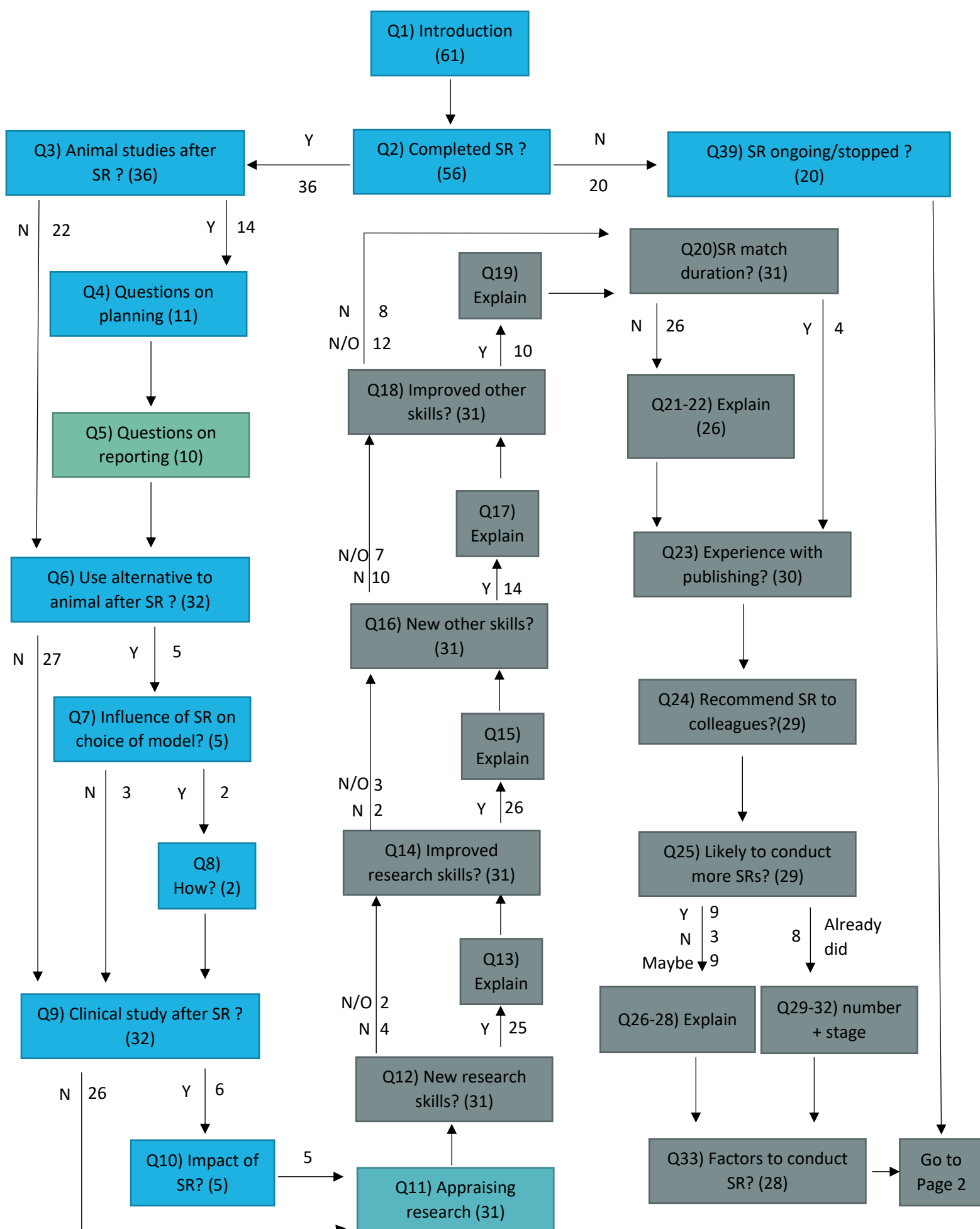

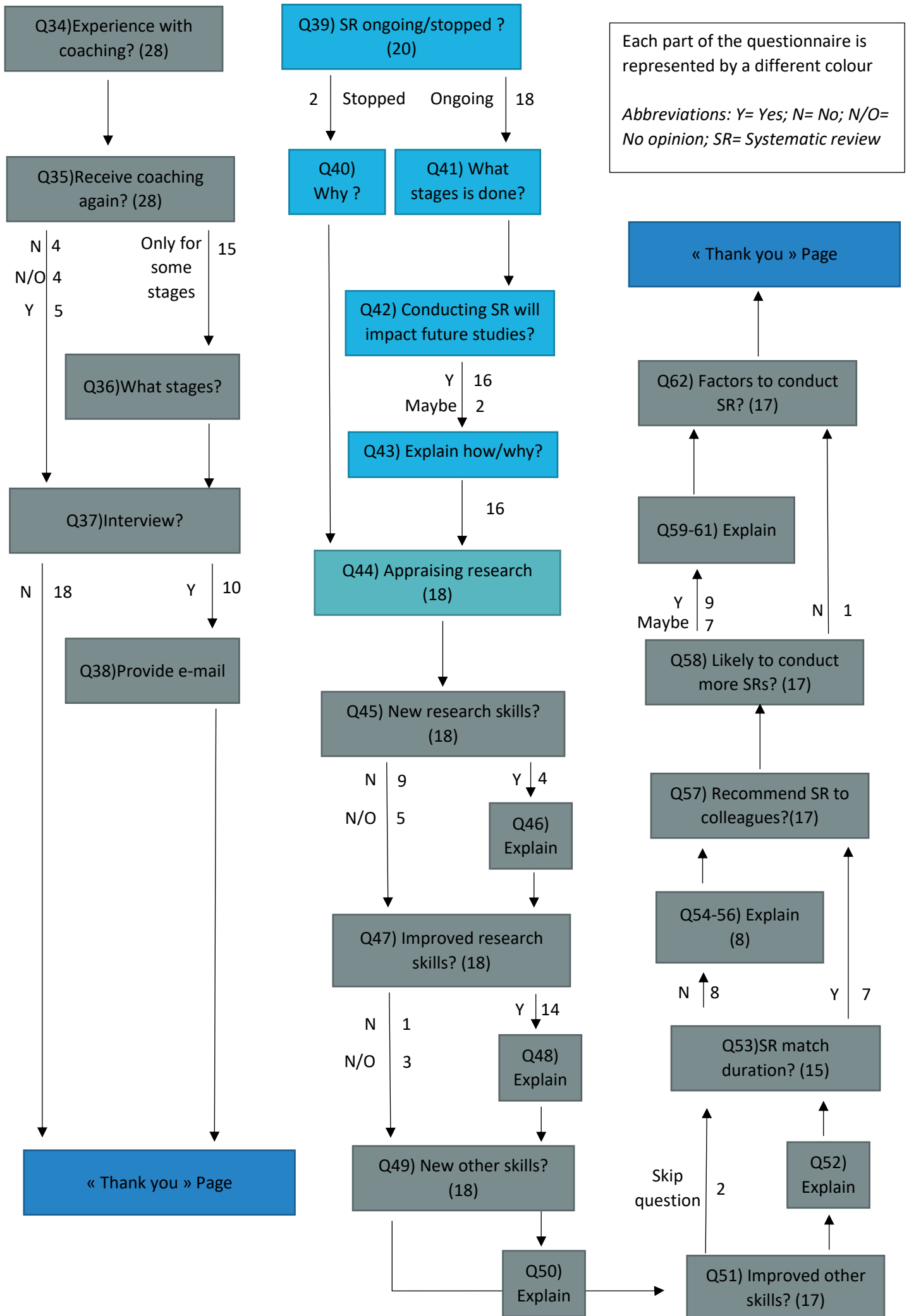

Supplement: S10 Appendix — (PDF) [file pone.0260619.s010.pdf]
