# Supplementary material for: The impact of conducting preclinical systematic reviews on researchers and their research: A mixed method case study
Source: PLoS One. 2021 Dec 13;16(12):e0260619. doi: 10.1371/journal.pone.0260619 (PMC8668092; doi:10.1371/journal.pone.0260619)
Supplement: S11 Appendix — (PDF) [file pone.0260619.s011.pdf]

S11 Appendix. Questionnaire results for questions on planning, designing and reporting animal experiments, and appraising research after conducting a preclinical SR.

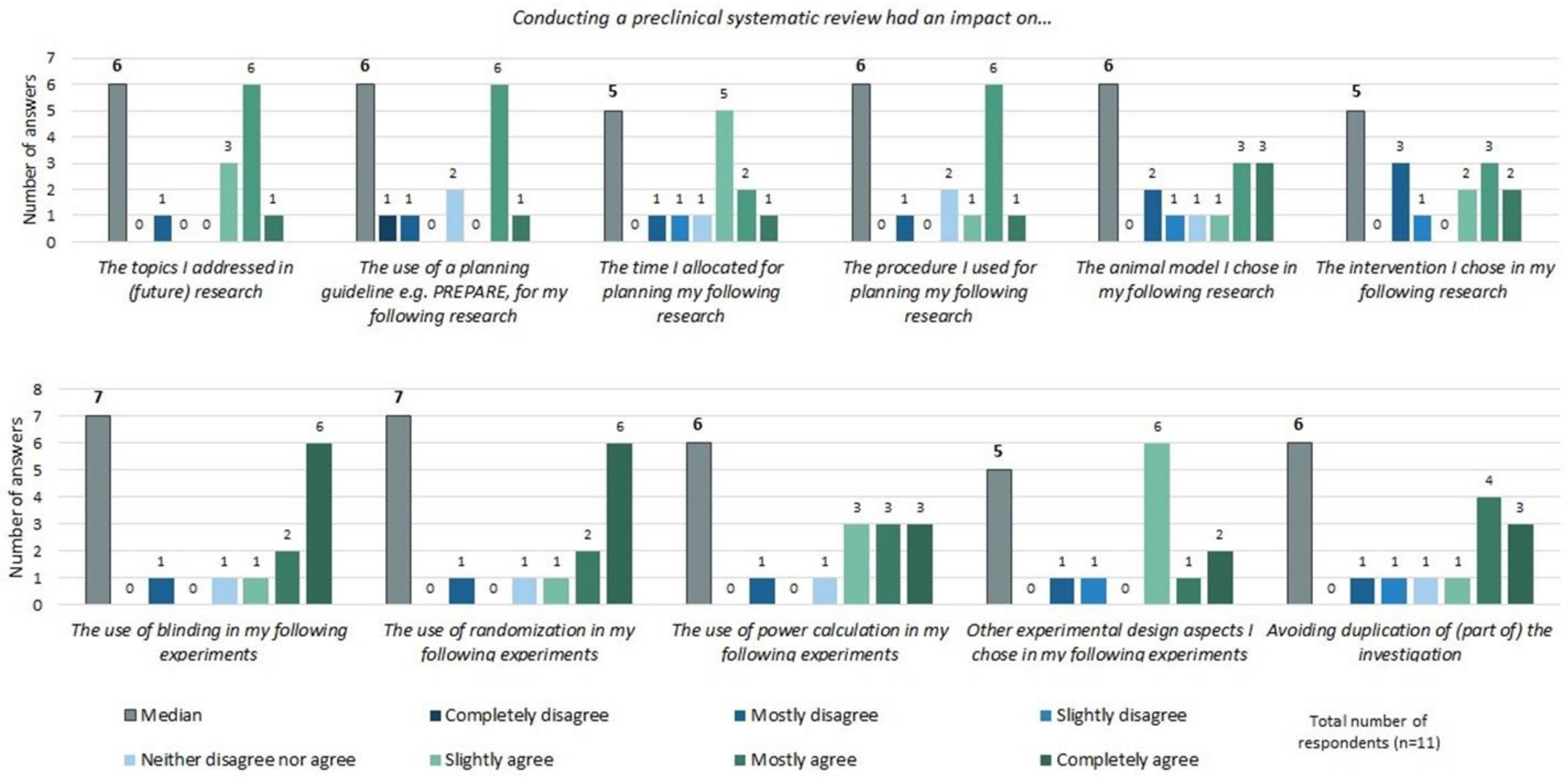

Fig 11.1. Overview of answers on planning and designing animal experiments after conducting a preclinical SR.

The scale used is from 1-7 (Seven points Likert scale), with 1 corresponding to completely disagree and 7 to completely agree. We show on the left (grey bar) the median for each question. The median shows the value (from 1-7) which was the most answered. The total number of respondents was 11.

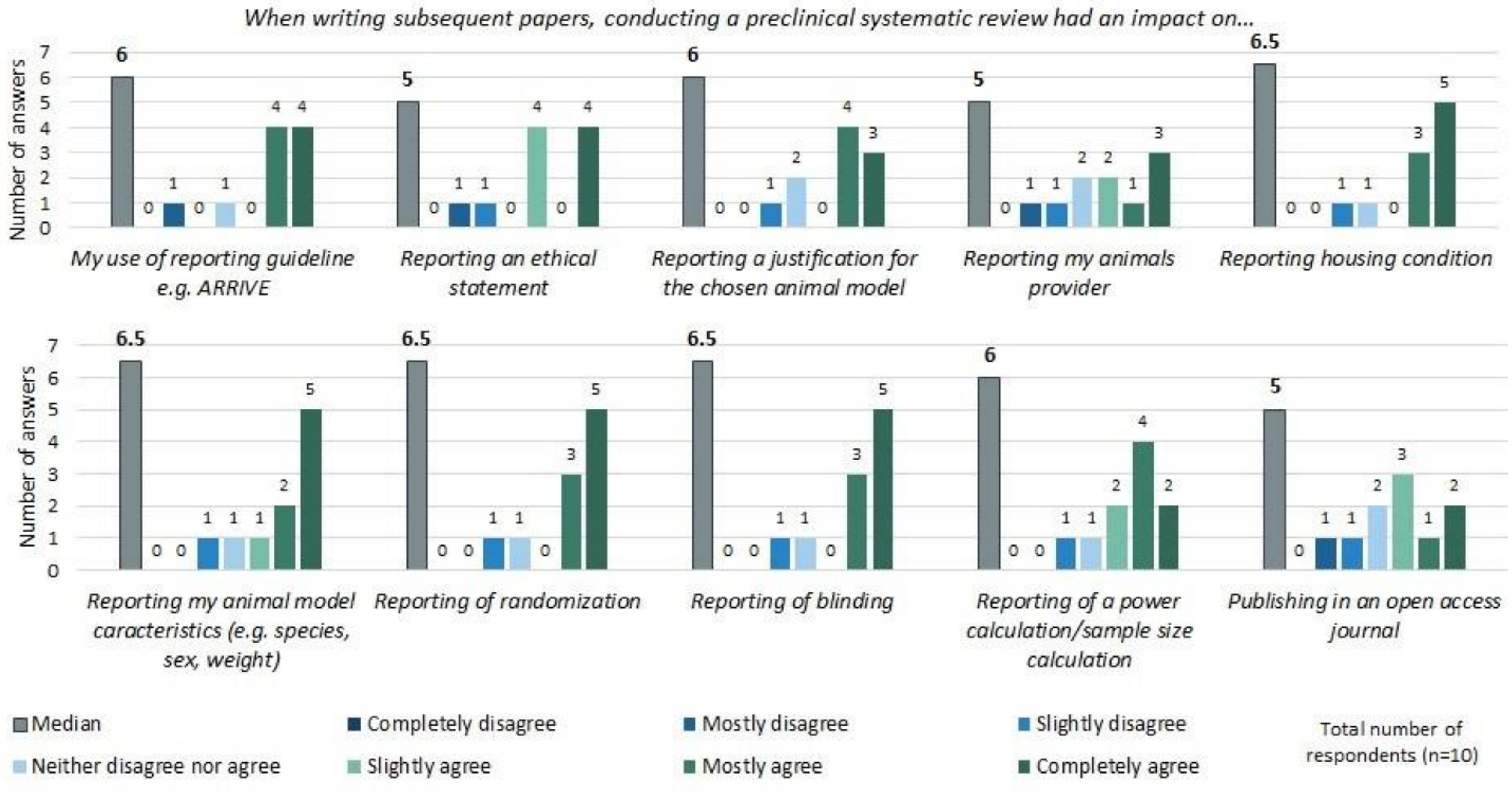

Fig 11.2. Overview of answers on writing about animal experiments after conducting a preclinical SR.

The scale used is from 1-7 (Seven points Likert scale), with 1 corresponding to completely disagree and 7 to completely agree. We show on the left (grey bar) the median for each question. The median shows the value (from 1-7) which was the most answered. The total number of respondents was 10.

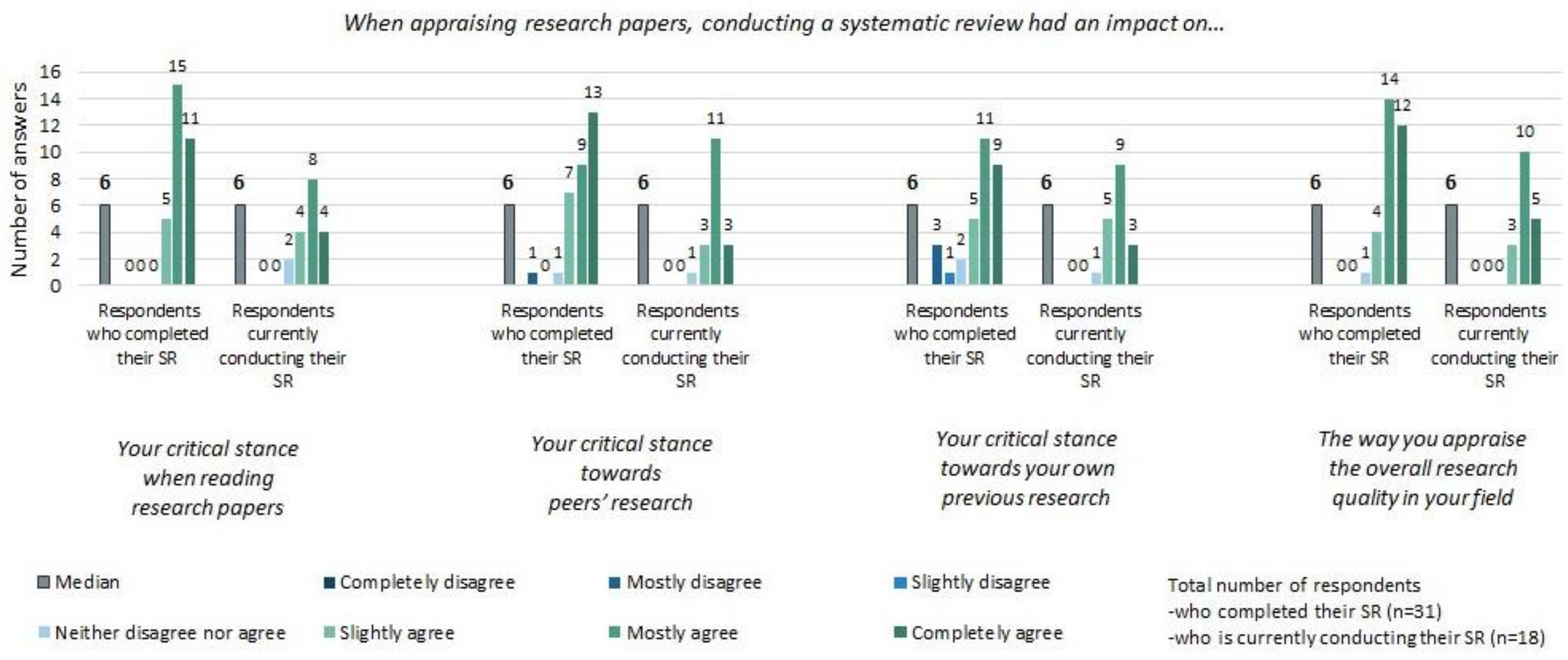

Fig 11.3. Overview of answers on appraising research.

This figure combines both groups of participants i.e., participants who have completed their SRs and participants who are currently conducting their SR. The first group is always presented on the left, and the second on the right. The scale used is from 1-7 (Seven points Likert scale), with 1 corresponding to completely disagree and 7 to completely agree. The median in grey shows the value (from 1-7) which was the most answered
