# Supplementary material for: The impact of conducting preclinical systematic reviews on researchers and their research: A mixed method case study
Source: PLoS One. 2021 Dec 13;16(12):e0260619. doi: 10.1371/journal.pone.0260619 (PMC8668092; doi:10.1371/journal.pone.0260619)
Supplement: S12 Appendix — (PDF) [file pone.0260619.s012.pdf]

S12 Appendix. Skills learnt and improved by performing preclinical systematic review.

Table 12. Overview of skills learnt and improved by participants after conducting (part of) their preclinical SR.

CATEGORIES	SKILLS	
**RESEARCH SKILLS DIRECTLY LINKED TO SR STAGES**	*Learnt and improved*<br>▪ Meta-analysis (and its interpretation)<br>▪ Risk of bias assessment, quality assessment and critical appraisal<br>▪ Creating a comprehensive search strategy<br>▪ Screening/ study selection and related software<br>▪ All stages of SRs	*Learnt*<br>▪ Data extraction and related software<br>▪ Subgroup analyses, publication bias<br>▪ Question phrasing<br>▪ Ordering results
**RESEARCH SKILLS FOR PLANNING/CONDUCTING SUBSEQUENT RESEARCH**	*Learnt and improved*<br>▪ Better experimental design of animal studies<br>-Including better methods for randomisation, blinding<br>-Awareness of models<br>-Awareness of field	*Improved*<br>▪ Better reporting/ writing skills<br>▪ Including proper controls<br>▪ Including patient participation
**CRITICAL APPRAISAL**	*Improved*<br>▪ Own critical appraisal<br>▪ Reviewing manuscripts<br>▪ Critical reading	
**INTERPERSONAL SKILLS**	▪ Project management and planning<br>▪ Persuading editors/ reviewers of the value of preclinical SRs<br>▪ Interdisciplinary work<br>▪ Communication and collaboration<br>▪ Training/ managing screeners and mentoring<br>▪ Acquiring a more structured way of thinking<br>▪ Consistency in repeated tasks	
