# Supplementary material for: The impact of conducting preclinical systematic reviews on researchers and their research: A mixed method case study
Source: PLoS One. 2021 Dec 13;16(12):e0260619. doi: 10.1371/journal.pone.0260619 (PMC8668092; doi:10.1371/journal.pone.0260619)
Supplement: Attachment — Submitted filename: PONE-D-21-16684_reviewer.pdf [file pone.0260619.s014.pdf]

PLOS ONE

The impact on research and researchers of conducting preclinical systematic reviews: a mixed methods case study

--Manuscript Draft--

**Manuscript Number:**	PONE-D-21-16684
**Article Type:**	Research Article
**Full Title:**	The impact on research and researchers of conducting preclinical systematic reviews: a mixed methods case study
**Short Title:**	The impact of conducting preclinical systematic reviews: a mixed methods case study
**Corresponding Author:**	Julia Menon, MSc<br>Interuniversity Cardiology Institute of the Netherlands: ICIN-Netherlands Heart Institute<br>Utrecht, NETHERLANDS
**Keywords:**	Research impact; Systematic reviews; case study; survey; interviews
**Abstract:**	Background: Systematic reviews (SRs) are a cornerstone of evidence-based medicine and have contributed significantly to breakthroughs since the 1980's. However, preclinical SRs remain relatively rare despite their many advantages. Since 2011 the Dutch health funding organisation (ZonMw) has run a grant scheme dedicated to promoting the training, coaching and conduct of SRs. This study focuses on this funding scheme to investigate the relevance, effects and benefits of conducting preclinical SRs on researchers and their research.<br><br>Methods: We recruited researchers who follow funded preclinical SR workshops and who conducted a SR with funded coaching. We gathered data using online questionnaires followed by semi-structured interviews. Both aimed to explore the impact of conducting a SR on researchers' subsequent work, attitudes, and views of their research field. Data-analysis was performed using Excel and ATLAS.ti.<br><br>Results: Conducting preclinical SRs had impact in two distinct ways. First, the researchers acquired new skills and insights, leading to a change in mindset regarding quality of animal research. The main impact was seen in the way participants planned, conducted and reported their subsequent animal studies, which were more transparent and of a higher quality than their previous work. Second, participants were eager to share their newly acquired knowledge within their laboratories and to advocate for change within their research teams and fields of interest. In particular they emphasised the need for preclinical SRs and improved experimental design within preclinical research, promoting these through education and published opinion papers.<br><br>Conclusion: Being trained and coached in the execution of preclinical SRs appears to trigger many beneficial changes which will impact the quality of preclinical research in the long-term. Our findings suggest that this ZonMw funding scheme is effective in improving the quality and transparency of preclinical research. Similar funding schemes should be encouraged in the future.
**Order of Authors:**	Julia M. L. Menon, MSc
	Merel Ritskes-Hoitinga
	Pandora Pound
	Erica van Oort
**Additional Information:**	
**Question**	**Response**
**Financial Disclosure**<br><br>Enter a financial disclosure statement that describes the sources of funding for the	Funding for this study was provided by the Netherlands Organisation for Health Research and Development (ZonMw) (https://www.zonmw.nl/en/), reference number 50009817. It was awarded to MRH.

Powered by Editorial Manager® and ProduXion Manager® from Aries Systems Corporation

work included in this submission. Review the submission guidelines for detailed requirements. View published research articles from *PLOS ONE* for specific examples.<br><br>This statement is required for submission and **will appear in the published article** if the submission is accepted. Please make sure it is accurate.<br><br>**Unfunded studies**<br>Enter: *The author(s) received no specific funding for this work.*<br><br>**Funded studies**<br>Enter a statement with the following details:<br>• Initials of the authors who received each award<br>• Grant numbers awarded to each author<br>• The full name of each funder<br>• URL of each funder website<br>• Did the sponsors or funders play any role in the study design, data collection and analysis, decision to publish, or preparation of the manuscript?<br>• **NO** - Include this sentence at the end of your statement: *The funders had no role in study design, data collection and analysis, decision to publish, or preparation of the manuscript.*<br>• **YES** - Specify the role(s) played.<br><br>* typeset	ZonMw, represented here by Erica van Oort, played a role in study design, management and coordination of responsibilities in planning and reviewing of the manuscript.
**Competing Interests**<br><br>Use the instructions below to enter a competing interest statement for this submission. On behalf of all authors, disclose any competing interests that could be perceived to bias this work—acknowledging all financial support and any other relevant financial or non-financial competing interests.<br><br>This statement is required for submission and **will appear in the published article** if the submission is accepted. Please make sure it is accurate and that any funding sources listed in your Funding Information	I have read the journal's policy and the authors of this manuscript have the following competing interests:<br><br>Julia Menon declares that she worked for the Department “Health Evidence” within the Radboudumc, in the same team as the coaches who provided training and support for the “knowledge infrastructure” module. Since February 2021, she has a paid position via ZonMw.<br><br>Merel Ritskes-Hoitinga, who supervised this study, is the head of the SYstematic Review Center for Laboratory (animal) Experimentation (SyRCLE) team.<br><br>Pandora Pound declared no competing interest.<br><br>Erica van Oort, who supervised this study, is project manager for ZonMw and is in charge of the “knowledge infrastructure” module, as part of the ZonMw MKMD programme.

Powered by Editorial Manager® and ProduXion Manager® from Aries Systems Corporation

later in the submission form are also declared in your Financial Disclosure statement.<br><br>View published research articles from *PLOS ONE* for specific examples.<br><br>**NO authors have competing interests**<br><br>Enter: *The authors have declared that no competing interests exist.*<br><br>**Authors with competing interests**<br><br>Enter competing interest details beginning with this statement:<br><br>*I have read the journal's policy and the authors of this manuscript have the following competing interests: [insert competing interests here]*<br><br>* typeset	However, we all sincerely declare that we did our utmost to remain impartial when conducting, analysing and supervising this study.
**Ethics Statement**<br><br>Enter an ethics statement for this submission. This statement is required if the study involved:<br><br>• Human participants<br>• Human specimens or tissue<br>• Vertebrate animals or cephalopods<br>• Vertebrate embryos or tissues<br>• Field research<br><br>Write "N/A" if the submission does not require an ethics statement.<br><br>General guidance is provided below. Consult the submission guidelines for detailed instructions. **Make sure that all information entered here is included in the Methods section of the manuscript.**	Participants were not patients, children, or vulnerable persons, and the topics addressed did not relate to their health, traumatic events or sensitive matters [35]. For these reasons, we did not seek approval of an Institutional Review Board as the topics addressed in both questionnaires and interview guides pose no risks to the participants, and in particular no risk of physical or mental harm. In addition, we performed several measures pertaining to anonymity, confidentiality, privacy, and informed consent, which complied with qualitative research standards and to the data protection act of 2018. All data were anonymised and assigned a random identification code. As noted above, an informed consent form was signed by interviewees. Interview recordings were deleted 16 weeks after the interview, and any mention of the participants' names, institutes, or any indicators that could threaten anonymity were omitted from the transcript. Lastly, only the primary investigator (JMLM) had access to the unblinded data.

Powered by Editorial Manager® and ProduXion Manager® from Aries Systems Corporation

**Format for specific study types**<br><br>**Human Subject Research (involving human participants and/or tissue)**<br>• Give the name of the institutional review board or ethics committee that approved the study<br>• Include the approval number and/or a statement indicating approval of this research<br>• Indicate the form of consent obtained (written/oral) or the reason that consent was not obtained (e.g. the data were analyzed anonymously)<br><br>**Animal Research (involving vertebrate animals, embryos or tissues)**<br>• Provide the name of the Institutional Animal Care and Use Committee (IACUC) or other relevant ethics board that reviewed the study protocol, and indicate whether they approved this research or granted a formal waiver of ethical approval<br>• Include an approval number if one was obtained<br>• If the study involved *non-human primates*, add *additional details* about animal welfare and steps taken to ameliorate suffering<br>• If anesthesia, euthanasia, or any kind of animal sacrifice is part of the study, include briefly which substances and/or methods were applied<br><br>**Field Research**<br><br>Include the following details if this study involves the collection of plant, animal, or other materials from a natural setting:<br>• Field permit number<br>• Name of the institution or relevant body that granted permission	
**Data Availability**<br><br>Authors are required to make all data underlying the findings described fully available, without restriction, and from the time of publication. PLOS allows rare exceptions to address legal and ethical concerns. See the PLOS Data Policy and FAQ for detailed information.	Yes - all data are fully available without restriction

A Data Availability Statement describing where the data can be found is required at submission. Your answers to this question constitute the Data Availability Statement and **will be published in the article**, if accepted.<br><br>**Important:** Stating 'data available on request from the author' is not sufficient. If your data are only available upon request, select 'No' for the first question and explain your exceptional situation in the text box.<br><br>Do the authors confirm that all data underlying the findings described in their manuscript are fully available without restriction?	
**Describe where the data may be found in full sentences. If you are copying our sample text, replace any instances of XXX with the appropriate details.**<br><br>• If the data are **held or will be held in a public repository**, include URLs, accession numbers or DOIs. If this information will only be available after acceptance, indicate this by ticking the box below. For example: *All XXX files are available from the XXX database (accession number(s) XXX, XXX.).*<br>• If the data are all contained **within the manuscript and/or Supporting Information files**, enter the following: *All relevant data are within the manuscript and its Supporting Information files.*<br>• If neither of these applies but you are able to provide **details of access elsewhere**, with or without limitations, please do so. For example:<br><br>*Data cannot be shared publicly because of [XXX]. Data are available from the XXX Institutional Data Access / Ethics Committee (contact via XXX) for researchers who meet the criteria for access to confidential data.*<br><br>*The data underlying the results presented in the study are available from (include the name of the third party*	All relevant data are within the manuscript and its Supporting Information files.

and contact information or URL).<br>• This text is appropriate if the data are owned by a third party and authors do not have permission to share the data.<br><br>* typeset	
Additional data availability information:	

Powered by Editorial Manager® and ProduXion Manager® from Aries Systems Corporation

Manuscript

Click here to access/download;Manuscript;The impact of conducting preclinical SRs _PLoS One.docx

The impact on research and researchers of conducting preclinical systematic reviews: a mixed methods case study

Julia M. L. Menon[a,b*], Merel Ritskes-Hoitinga[a,c], Pandora Pound[d], Erica van Oort[e]

[a] Systematic Review Centre for Laboratory (animal) Experimentation (SYRCLE), Department for Health Evidence, Radboud University Medical Center, Nijmegen, the Netherlands

[b] Preclinicaltrials.eu, Netherlands Heart Institute, Utrecht, The Netherlands

[c] AUGUST, Department for Clinical Medicine, Aarhus University, Denmark

[d] Safer Medicines, Kingsbridge, United Kingdom

[e] ZonMw (Netherlands Organisation for Health Research and Development),The Hague, The Netherlands

*Corresponding author: Preclinicatrials.eu, Netherlands Heart Institute, Moreelsepark 1, 3511 EP Utrecht, Netherlands. E-mail: Julia.menon@heart-institute.nl

Abstract

Background: Systematic reviews (SRs) are a cornerstone of evidence-based medicine and have contributed significantly to breakthroughs since the 1980's. However, preclinical SRs remain relatively rare despite their many advantages. Since 2011 the Dutch health funding organisation (ZonMw) has run a grant scheme dedicated to promoting the training, coaching and conduct of SRs. This study focuses on this funding scheme to investigate the relevance, effects and benefits of conducting preclinical SRs on researchers and their research.

Methods: We recruited researchers who follow funded preclinical SR workshops and who conducted a SR with funded coaching. We gathered data using online questionnaires followed by semi-structured interviews. Both aimed to explore the impact of conducting a SR on researchers' subsequent work, attitudes, and views of their research field. Data-analysis was performed using Excel and ATLAS.ti.

Results: Conducting preclinical SRs had impact in two distinct ways. First, the researchers acquired new skills and insights, leading to a change in mindset regarding quality of animal research. The main impact was seen in the way participants planned, conducted and reported their subsequent animal studies, which were more transparent and of a higher quality than their previous work. Second, participants were eager to share their newly acquired knowledge within their laboratories and to advocate for change within their research teams and fields of interest. In particular they emphasised the need for preclinical SRs and improved experimental design within preclinical research, promoting these through education and published opinion papers.

Conclusion: Being trained and coached in the execution of preclinical SRs appears to trigger many beneficial changes which will impact the quality of preclinical research in the long-term. Our findings

suggest that this ZonMw funding scheme is effective in improving the quality and transparency of preclinical research. Similar funding schemes should be encouraged in the future.

Introduction

Keeping up to date with the literature can be challenging due to the vast number of new articles published every year. Scholarly peer-reviewed journals produce over 3 million articles per year, with a 56% increase since the last decade [1, 2]. Consequently, researchers, policymakers and healthcare providers require a way to systematically identify and evaluate literature on a specific topic. For 40 years, systematic reviews (SRs) have provided a powerful way of achieving this within clinical research-. SRs follow clear and defined steps, and aim to provide a synthesis as well as a critical assessment, of all the available relevant evidence [3]. Such syntheses enable researchers to identify research gaps and help future decision making in practice and policy. The methodology of SRs emerged from the evidence-based medicine paradigm of the 1980's [4, 5]. Today, they are a cornerstone of evidence-based medicine, with 30,000 SRs protocols being registered as of 2017 [6]. However, despite their advantages, SRs are struggling to achieve similar status in the preclinical field [7]. A lack of knowledge, skills, or awareness of their value may be to blame. The first preclinical SRs began to appear in the early 2000s, a decade or so after the clinical SR standards were established [8]. Despite the slow start, preclinical SRs have been shown to increase transparency, avoid unnecessary duplication and help identify and improve poor reporting and poor study design [7, 9, 10]. They can be used retrospectively to throw light on clinical trial data, or prospectively to prepare for new clinical and preclinical studies, and they may be used to guide future (translational) research [7, 9, 10].

Interest in preclinical SRs is slowly growing within academia and amongst other stakeholders [11-15]. Since 2011, the Netherlands Organisation for Health Research and Development (ZonMw) has invested in education and coaching in preclinical SRs , through their funding programme "More Knowledge with Fewer Animals" (Meer Kennis met Minder Dieren in Dutch, or MKMD) [15]. The main goal of this funding programme is to promote and implement the adoption of animal-free research methods by

funding new animal-free (human based) innovations and encouraging the use of already existing alternatives . The programme consists of several modules, each with a different focus [16]. The Knowledge infrastructure module focuses on providing preclinical SR education and coaching via workshops and training, and on promoting open access publishing of (negative or neutral) results [16]. Since its formation, 22 SR workshops have been organized within the Netherlands (over the period 2013-2020) and many participants have subsequently enrolled to conduct a coached preclinical SR.

While the benefits of clinical SRs are beyond doubt, the impact of conducting a preclinical SR on preclinical researchers and their research field remained unknown. Hence, to investigate these effects, we evaluated the impact of ZonMw funded preclinical SRs on researchers and (their) research.

Research questions

What impact does conducting a preclinical SR have on researchers, their research and their field?

1. What impact does conducting a preclinical SR have on a researcher in terms of planning, conducting, reporting and appraising their research projects?

2. What impact does conducting a preclinical SR have on a researcher's views about research, both their own and their field more generally?

3. What impact do preclinical SRs have on preclinical research in general (e.g., quality, reproducibility, transparency, accessibility)?

Theoretical framework

This project is a research impact case study aiming to evaluate the impact of an intervention (conducting preclinical SRs) on individuals (researchers), products (their research), and environment (their research field). Therefore, it relies on theories and knowledge of research impact assessment. An in-depth description of our theoretical framework can be found in the original ZonMw internal report made for this case study, available at: https://www.zonmw.nl/nl/actueel/nieuws/detail/item/systematisch-literatuuronderzoek-vervangt-vermindert-en-verfijnt-proefdieronderzoek/.

In short, research impact assessments rely on the evaluation of research impacts, defined as "contributions that research makes to the economy, society, culture, national security, public policy or services, health, the environment, or quality of life, beyond contributions to academia" [17]. These evaluations tend to be conducted to address one or several of the 4As: Advocacy, Accountability, Analysis and learning, and Allocation [18, 19].

Overall, research impacts will lead to demonstrable and beneficial changes in behaviours, beliefs, and practices [20]. Consequently, these evaluations usually assess all directions and categories of impacts in a structured and high-quality manner [21]. This may include the use of complex, time-intensive frameworks, including impact pathways to identify or foresee how research projects create (expected) impacts, for example societal or environmental impact of a research project [22, 23]. Since impact evaluations began to be conducted by universities in the 1970's and 1980's, a plethora of frameworks have been proposed, with different scopes, aims, and assets [22-24]. Each proposes a specific approach to assessing research knowledge and research quality, and to measuring impacts [25]. Well-known frameworks include the Canadian Academy of Health Sciences Preferred Framework, the National Institute of Health Research Dashboard, or the Excellence in Research for Australia [23, 26, 27]. Such

large endeavours were considered out of scope for our current project. Therefore, we chose two smaller frameworks to structure our study, namely 1) the research impact framework, and 2) the behaviour wheel of change.

The research impact framework was developed in 2007 by Kuruvilla et al., as a checklist to guide researchers in selecting and evaluating the impacts of their work and interventions [28]. It highlights four areas of impacts: 1) research-related impacts, 2) policy impacts, 3) service impacts, and 4) societal impacts. We focused on the first area and designed our study using the seven categories it provides, which range from "providing data about a problem", "study replication", "innovation of new methods", to subtler impacts such as "becoming a member of a scientific society". All seven categories are available in detail in S1 Appendix.

The behaviour wheel of change created by Michie et al., in 2011, presents the idea that behavioural change occurs as a result of good predisposition, interventions, and policies (which enable or support the particular intervention and are created [in part] by responsible authorities) [29](See S2 Appendix). These good pre-dispositions are capability, opportunity, and motivation as supported by the COM-B system - a behavioural change theory [29, 30]. Within the intervention covered by this framework, three types corresponded to our case study: education, training, and enablement.

As a result, we hypothesise a pathway by which our intervention (conducting preclinical SRs) in a set context (supported by ZonMw's workshops, coaching and funds) would create impacts (for researchers and their research) (Fig 1).

Fig 1: Visual representation of the current hypothesis on impacts

Materials and Methods

This study complies with the standard for reporting qualitative research and the consolidated criteria for reporting qualitative research [31, 32]. Checklists are available in S3 and S4 Appendix.

Investigator characteristics and reflexivity

The primary investigator is a research assistant with a Master's Degree in Science, Innovation and Management (Radboud University). She has experience in preclinical SRs and qualitative research via her Master's Degree and two long-term Master's internships. She had no relationship with any of the participants and had not taken part in the training or coaching provided by the funding scheme being studied, although she had taken part in one of the workshops. Participants were not aware of her personal goals or personal thrive to conduct this research project.

Her interest in preclinical SRs and qualitative research led her to conduct this research project. She was supported and/or supervised by a research fellow with a background in ethics and philosophy, a professor in Evidence-Based Laboratory Animal Science and a ZonMw project manager, all of whom helped create a robust framework for the project.

Context

This study uses a mixed-method approach combining questionnaires and semi-structured interviews. We focused on researchers who had followed ZonMw's workshops and who (had) received coaching to perform their own preclinical SR. This enabled us to get a snapshot of the preclinical SR field and evaluate fairly the experience of these researchers within a given frame.

Sampling strategy

We used purposive sampling to select our participants. The target population for the questionnaires comprised researchers who had participated in a ZonMw workshop and who had either started or completed a preclinical SR with coaching. Those who had completed their SRs were approached to take part in an interview, to evaluate the impact of the SR on their subsequent research.

Recruiting participants

Participants were contacted by e-mail and sent a reminder two weeks after the first invitation (both the invitation and reminder are available in S5 Appendix). All e-mail addresses were obtained from the information giving during coaching . Some email addresses were inactive and participants could not be traced despite extensive efforts.

Due to the nature of this project, we did not aim to achieve data saturation, but rather to collect as much data as possible within the given timeframe.

Data collection methods

The questionnaires and semi-structured interviews were considered complementary, with the interviews providing greater detail than the questionnaires. The questionnaires provided quantitative information about the impacts of preclinical SRs, while the interviews provided more in-depth data. Data collection occurred for a period of 6 weeks (23/07/2020 - 04/09/2020), with the online questionnaires being available for one month (23/07/2020 - 23/08/2020). Data analysis was performed for a period of almost one month (24/08/2020-18/09/2020).

Online questionnaires

We designed and uploaded the questionnaire onto "Questionpro" (https://www.questionpro.com/), a free questionnaire platform. Our questionnaire comprised of both closed (dichotomous, multiple-choice or scaled) questions and open-ended questions. For rating questions, seven-point Likert scales were chosen (to avoid the bias created by using five-point Likert scales), with the extremities being "completely disagree" and "completely agree" [33]. The participants were divided into two groups; the "SR completed group" and the "SR started group". The groups were kept separate by skip logic. For the former group, five categories of impact were addressed 1) designing and planning experiments, 2) writing manuscripts, 3) appraising research, 4) skills after conducting the SR, and 5) experience with conducting a SR (including, but not limited to, publishing experiences and wishing to perform further SRs). For the latter group, only points 3, 4 and 5 were evaluated. At the end of the questionnaires, researchers in the "SR completed" group were also invited to participate in a semi-structured interview on the same topic. The full questionnaire is available in S6 Appendix.

Semi-structured interviews

Researchers willing to participate in an interview were sent an informed consent form by e-mail and were given the opportunity to ask questions before signing (available in S7 Appendix). They were ensured that they could withdraw from the study at any point without any consequences. The informed consent form was based on the World Health Organisation informed consent form template for qualitative studies (https://www.who.int/ethics/review-committee/informed_consent/en/).

The semi-structured interviews lasted about one hour and were conducted by teleconference using GoToMeeting. Only the participant and researcher were present on the call. Interviews were structured using a list of open questions on the participant's experience with their SR and the impacts they felt it had on their research, their attitudes, and their research field. (The interview

guide can be found in S8 Appendix). Field notes were written during and/or after the interviews and were anonymised

Data processing & Data analysis

Questionnaire data were exported from Questionpro to Excel. The 'SR completed' and 'SR ongoing' groups were analysed separately. Data analysis consisted of frequency counts for close-ended questions (with calculations of median and means), while open-ended questions were subject to content analysis. All analysis was conducted by one reviewer (JMLM).

The interviews were video recorded via GoTo Meeting, converted into mp3 files using VLC player, and subsequently transcribed verbatim using Express Scribe Transcription Software. All recordings were anonymised; random numbers were acquired from www.random.org. Transcripts were not returned to participants for comments and/or corrections to not bias their first answers (as this evaluation is performed for a funding agency). Thematic analysis of the transcripts was conducted by one reviewer (JMLM) in Atlas.ti (Version 8.4.15.0), with themes derived from the data and some coming from the Research impact framework [28, 34]. The coding tree and code organisation can be seen in S9 Appendix. Feedback options were not included for participants' on findings of either questionnaires or interviews.

Ethical concerns pertaining to human subjects

Participants were not patients, children, or vulnerable persons, and the topics addressed did not relate to their health, traumatic events or sensitive matters [35]. For these reasons, we did not seek approval of an Institutional Review Board as the topics addressed in both questionnaires and interview guides pose no risks to the participants, and in particular no risk of physical or mental harm. In addition, we performed several measures pertaining to anonymity, confidentiality, privacy, and informed consent,

which complied with qualitative research standards and to the data protection act of 2018. All data were anonymised and assigned a random identification code. As noted above, an informed consent form was signed by interviewees. Interview recordings were deleted 16 weeks after the interview, and any mention of the participants' names, institutes, or any indicators that could threaten anonymity were omitted from the transcript. Lastly, only the primary investigator (JMLM) had access to the unblinded data.

Techniques to enhance trustworthiness

Thorough piloting was performed for both questionnaires and semi-structured interviews by several researchers knowledgeable in SRs and coming from different backgrounds and career levels namely research assistant, PhD student, (associate) professor (n=7).

Triangulation was performed in the planning and designing of questionnaire and interview guide between the main investigator and (most of) the co-authors.

Results

Response rate

We began with 99 potential participants, of whom were able to contact 95. Sixty-one participants started our questionnaire, and 45 completed it (i.e., 16 drop-outs), giving a response rate of 64.2% and a completion rate of 47.4%. An overview of participants per phase is available in Fig 2.

From the 61 participants, 36 belonged to the “SR completed” group (i.e., published or submitted a manuscript of their preclinical SR). In comparison, 18 participants belonged to the “SR ongoing” group (i.e., still in the process of conducting their SR). Two participants did not complete their SRs due to time constraints. And the 7 remaining participants dropped the questionnaire before answering the

question about the state of their review. Ten participants agreed to participate in interviews; eight interviews were eventually conducted due to unavailability of two researchers.

Fig 2. Number of participants per phase

Abbreviation: SRs: Systematic Reviews, NR: Not reported

Questionnaires

An overview of the questions and the number of respondents for each question can be found in S10 Appendix. The (anonymised) results of the questionnaire are available in S13 Appendix.

Impact on planning, designing, and writing research projects

Within the SR completed group (n=36), 14 participants went on to perform primary animal studies after completion of their SRs, 5 performed preclinical research using alternatives to animals and 6 performed clinical studies.

All participants who performed animal studies after their SRs answered questions about planning, designing, and reporting these studies. Most respondents agreed with the statements asked, as illustrated by the median of each question. The average median for Fig 3A, (planning/conducting experiment) is 5.36/7, while the average median for Fig 3B (reporting) is 5.90/7.

Fig 3: Median answers on planning, designing, and writing subsequent research after conducting a preclinical SR.

Both graphs show medians per question of a 7 Likert scale. On this scale, 1 corresponded to completely disagree and 7 to completely agree. Median with values of 5 or above means that respondents agreed with the statements.

A: median answers on planning and designing subsequent research after conducting a preclinical SR (n=11)

B: median answers on writing subsequent research papers after conducting a preclinical SR. The total number of respondents was (n=10).

We found that conducting preclinical SRs impacted the way participants planned their future studies, in that they increased the use of planning guidelines and time allocated for planning, the use of power calculations to ensure statistical validity, and they contributed to avoiding unnecessary duplication. Moreover, conducting a preclinical SR also impacted the way participants performed their subsequent animal study ,for example in terms of the animal model and intervention chosen, and use of blinding and randomisation. Similar results can be seen with regard to reporting. Conducting preclinical SRs strongly impacted the quality of reporting, including but not limited to increasing the use of reporting guidelines, appropriate reporting animal characteristics, housing conditions and methods (e.g., blinding and randomisation).

Altogether, the results suggest that conducting a preclinical SR improved the quality of participants' subsequent animal studies through better and more thoughtful planning and conduct, as well as improved transparency as a result of better reporting of information and methods. (Detailed answers for each question can be seen in Fig 11.1 and 11.2 in S11 Appendix).

The SR ongoing group (18 participants) answered less detailed questions about their future research. However, all felt that conducting their preclinical SR would influence the way they would conduct and report their next experiment. Some suggested it would impact the model they would choose, or the design of future *in vitro* studies, or would highlight data gaps for clinical research. Some participants felt there would be an impact on their field too. The following quotes illustrate these themes:

> *"Through the systematic review, we understand that the quality of currently published animal study reports is very poor, in particular, reporting randomisation and blinding. This reminds us*

to pay more attention to those aspects when designing, implementing and writing our own study." - Respondent 75156050

"This study aims at investigating the best animal models that result in a similar outcome to human X. […] Based on the findings of this study, I'd use the most robust models that will result in the reduction of the use of animals. I'm also confident that people who read this article will employ a similar approach and modify the design of their studies, thus overall, I think this study will have a big impact in the field" – Respondent 76823076

Of the five people who performed research projects using non-animal methods, two affirmed that their SR had positively influenced their choice of approach. The other three had already decided to use a non-animal approach prior to conducting their SRs. For the six who performed clinical research projects, three stated that the SR had impacted their clinical research; one with respect to the questions they chose to address, another in terms of translating preclinical to clinical research, and the third in terms of highlighting the need to investigate the role of a specific mutant phenotype

Impact on research appraisal

All participants answered questions about appraising research, regardless of the stage of their SR. The aim of these questions was to determine the effect of conducting a preclinical SR on the way they conducted appraisals.

Overall, the results were quite positive and were equal across groups (Fig 4). Few participants disagreed with the statements (data in Fig 11.3 in S11 Appendix). According to our findings, the critical stance of participants was sharpened by their experience with the preclinical SR, for both reading and assessing papers or research projects, and regardless of where that research comes from.

Fig 4: Median answers on appraising research and critical stance after conducting (part of) a preclinical SR

Both graphs show medians per question of a 7 Likert scale. On this scale, 1 corresponded to completely disagree and 7 to completely agree. Median with values of 5 or above means that respondents agreed with the statements. The total number of participants was 31 for the "Completed SR" group, and 18 for the "Ongoing SR" group.

Skills

Participants from both groups stated that they gained new skills or improved existing skills in the process of conducting their preclinical SRs. Some skills are exclusive to research (e.g., meta-analysis skills, academic writing, critical appraisal), while others go beyond research (e.g., collaboration, negotiating with editors, interdisciplinary working). We identified and categorised four main types of skills: 1) research skills directly linked to the SR stages, 2) Research skills for planning/conducting subsequent research, 3) Critical appraisal, and 4) Interpersonal skills (Table 12 in S12 Appendix).

Experience with their preclinical SRs

Even though many participants underwent extensive delays while conducting their reviews, most considered it likely that they would want to conduct another SR or advise colleagues and peers to conduct a preclinical SR. Numbers wise, 34/46 participants mentioned that they would be likely to conduct another SR (18 "yes" and 16 "maybe"), while 45/46 would potentially recommend colleagues to do their own SRs (37 "yes" and 8 "maybe"). Eight participants from the completed SR group had already conducted a second SR after completing their first coached review, illustrating how useful they considered this. Additionally, in the SR completed group, 20/28 participants reported that they would like to receive similar coaching for their next review. Fifteen of these wanted a more personalized approach, with a focus on some stages of the SR, while 5 wanted coaching for the whole process.

Interviews

The interviews afforded greater insight into the sort of impact that conducting a preclinical SR has on both researchers and their research, as well as how this happens. We identified that impacts occurred in two steps and influenced three given levels (Fig 5).

Fig 5: Process and steps by which conducting preclinical SRs result in impact for researchers and research at different levels

First, impacts occur at the level of the researchers; conducting the SRs enable an evolution in participants' thinking, providing them with important insights and skills. This first step leads researchers to step back from their usual point of view and triggers the realisation of specific issues and shortcomings in their own work, research field, and science community.

Second, impacts on research can occur now that a new mindset has developed. Researchers actively modify their activities 1) relative to their own work (*lab level*), 2) regarding how they appraise their field and advocate for change (*field level*), and 3) promoting change on a broader scale (*science community level*).

Both steps and levels of impact will be further explained and illustrated with empirical data in the upcoming sections.

Step One. Impacts on researchers: a change in mindset

Interviewees were from various academic backgrounds and fields. However, they achieved similar insights, skills, and realisations as a result of conducting their preclinical SR.

Even though most respondents were already aware of the reproducibility crisis, conducting their review brought them face to face with poorly designed and/or poorly reported studies. This triggered

realisation, not only about the poor quality of animal studies in their field, but about their own past mistakes:

> *"I was quite shocked how poorly designed some studies are, while still being published in a high impact journal" – ZonMw 72*

> *"I had two medical students, and they helped me, also during the risk of bias, and they were like 'how researchers can be so stupid, they are so stupid', and I was like 'well, I think I did this too'." – ZonMw 23*

The 'penny dropped' for most participants during the risk of bias assessment and data extraction stage, as many mentioned they were quite 'frustrated' due to unclear or missing information. This eye-opener on quality was confirmed when participants were unable to interpret studies properly or when these poor-quality publications impaired their final analysis.

> *"We had a lot of data, the amount was more than enough but because the quality, I would say, of the studies is pretty low and sometimes you can't find the right information, it's harder to draw conclusions."– ZonMw57*

As a result, participants reflected on their past flaws and learned how to prevent such mistakes in the future. For some, these issues made them rethink their activities in the preclinical field, their reasons for using animals and how they performed experiments:

> *"There's a lot of bias that could be introduced in a model and having done it myself, I really realised that it's really pitiful of doing these experiments if you don't do them in the proper way." – ZonMw84*

> *"It made me really more aware of why you [would] want to use animals and in what way. And even though in my own research I would want to do it in a good way, I saw that we also have flaws, and it made me more aware of what you're actually doing when you're doing animal research"- ZonMw57*

Despite insufficient quality or lack of evidence, most participants were able to draw conclusions and gain insights. We organised these insights under the four following categories: 1) increased awareness of their own field, 2) identification of data gaps and topics for new primary studies, 3) discovery of heterogeneity and incoherencies, and 4) discrediting or confirming theories.

1) *"You retrieve papers that you were not aware of even though you're quite familiar with literature on the topic, you will always find new things" – ZonMw80*

2) *"I really have more, much more transparent ideas about what studies are missing, and need to be done" – ZonMw23*

3) *"What was striking for this model is that everybody in the field was using the model in a different way." – ZonMw84*

4) *"We did encounter unexpected findings, which we couldn't even explain why it was the case. And it really contradicts some basic theories of (field of interest), of how our intervention works." – ZonMw 91*

Overall, participants were convinced about the value of preclinical SRs, were positive about their experiences of the funding scheme and the coaching. Moreover, many participants admitted that they had underestimated the difficulty of the SR process, and were glad to have received proper guidance.

"I had underestimated really the efforts needed to make a strong and a good systematic review" – ZonMw72

"I really felt I needed this help" – ZonMw23

Step Two. Impact on research (lab level)

Research impacts were identified within the participants' teams and labs and were categorised as

follows: 1) direct impacts on experiments, planning, conducting, reporting, appraising research; execution of other types of research, switching fields, conducting meta-research, and 2) advocacy for better research within their teams, promoting networking and collaboration.

The first category of impact was frequently mentioned in the questionnaires. Similar outcomes were reported in the interviews, namely that the planning, conduct and reporting of research projects were performed better after (or while) conducting the SR. The participants were unanimous about the change and progress they experienced thanks to their review:

> *"I was just much more mindful about the blinding, randomisation, the sources of bias. We put enormous amount of efforts into doing that properly" – ZonMw91*

> *"A lot of details would be missing if I hadn't done this (systematic review), cause now I know how much is missing in the studies" – ZonMw23*

Aside from doing new experimental research differently, participants' SRs fuelled ideas for other types of studies in meta-research and non-animal approaches. For some of them, the SR was a game-changer which led them to switch fields. Some now pursue their career in human research, some in meta-research (where they focus on SR, meta-analysis, or the development of further tools) and some have distanced themselves from academia to work towards improving the system:

> *"Step by step, you can do a lot of research in humans that is also very helpful. I did a lot of animal experiments and yeah (pause). I'm not going to do that anymore; I'm not going back to animal experiments" – ZonMw84*

> *"I realised then that open science is what I'm interested in. That's also why I wanted to switch to a different working environment. Not doing research myself anymore that much but actually working on trying to improve the system" – ZonMw57*

The second category of impact emphasises our participants' enthusiasm about sharing their newly acquired knowledge with colleagues and peers. Some passed on their knowledge by teaching their new skills to students or by advising colleagues on how to improve their research, as the following quotes show:

> *“I had this little extra time to teach students how to do it, and it becomes part of their package as well. Because in reality there's nothing about this type of science in education, and that's such a shame. Experimental design, statistics, and so, it's completely missed in education, and I feel that's what I want to teach my students, how to do good science” – ZonMw91*

> *“I also used this to give presentations and make my colleagues at the department aware of how they should perform animal experiments. So, I try to implement this guideline of ARRIVE also in our department.” – ZonMw84*

However, such improvements at a team or individual level may require changes from higher up in organisations. Several interviewees mentioned how supervisors’ lack of familiarity with SRs could impede their execution and the transfer of skills and knowledge within a team. The fact that some professors and supervisors realised the value of SRs by the time they had completed the SR does, however, provide hope.

> *“I have an assertive personality. […] But many other people that are not as assertive might just not do a systematic review in the first place because of how many criticisms there is from the more managerial position in the department. Meaning professors themselves don't understand, then why should a PhD student do it?” – ZonMw91*

Resistance to change remains in the lab, both towards acceptance of preclinical SRs but also to other new methods that can improve research quality. Lab conventions, resistance from colleagues and supervisors, time pressure, funding and competition were the main obstacles experienced by the participants.

Step Two. Impact on research (field level)

The two important ways by which our participants impacted their field with their preclinical SR were 1) by dissemination of their findings, and 2) by promoting the value of SRs to improve the quality of research, for example, by writing an opinion paper.

The usual way of disseminating results is via publications and conferences. According to interviewees' experiences, publishing preclinical SRs is no easy business. Some participants were stuck between finding journals that would accept their SRs or, once found, had to face repeated rebuttals. Moreover, the opinions of peer reviewers were quite influential in the editorial process. Responses about peer reviewers' input were mixed; some participants had a generally negative experience with peer reviewers who did not understand the SR process or the value of SRs, while others received beneficial input that improved their SR:

> *"This is I think the biggest frustration of the whole process; that the journals and the reviewers really underestimate the significance, the work, that is in it.[…] they said no 'you have to do a letter to the editor, it's not worth an original article' – ZonMw23*

This dissonance directly echoes the lack of acceptance, support and education regarding preclinical SRs as mentioned earlier in "Step Two. Impact on research (lab level)". Nevertheless, once in the open, preclinical SRs can influence a field, as shown by the two examples in box 1. Of course, publication does not ensure that the SR will be read, accepted, or that the recommended changes will be implemented by peers. Some participants had frustrating experiences, discovering that the insights from their SR had not (yet) made their field budge one bit.

Box 1: Example of field impacts

EXAMPLE 1

- A participant discovered that the mode of drug administration significantly impacted the results of an intervention, both in clinical and in preclinical experiments. With this issue identified, there could be a change on how this particular intervention is administered across the whole field.

*"With regard to the patient's studies, was that everybody just depleted these XX and saw what happened to XX, and then said, 'oh XX are bad' or 'oh XX are good'. But our review made I hope, make people realise when it gets published that it totally depends on **how** you administer your agent to deplete your XX. So, you can't really say 'oh they are good' or 'they are bad' because it depends totally on how you do it." – ZonMw23"*

EXAMPLE 2

- A participant's SR triggered a whole series of new SRs in the same direction as their own; by showing their field the value and insight a preclinical SR can have, other researchers began performing their own preclinical SRs.

"People really loved the paper and found it really informing to have a first good overview. And then, multiple research groups actually 'copy pasted' our paper, but put another cell type than the cell type we used […] for the research field it was good, because everybody started to systematically assess all the evidences for certain cell lines"- ZonMw15

To press for change, certain participants chose an innovative route: some wrote opinion papers or letters to reach peers and editors, while some others took the matter a step further by creating field-designed tools (e.g., apps and websites useful to their field).

> *"We wrote a paper in a XX journal for animal technicians, and describing all the differences that everybody does with the model. […] also concerning housing and all kinds of things that are important for animal studies. […] I also wrote a paper for the XX organisation, about the use of animals and the 10 sorts of pitfalls, mistakes about experimental models" – ZonMw84*

Step Two. Impact on research (science community level)

Impacts at a broader level were also identified, i.e. increased awareness and acceptance of preclinical SRs, which might then (hopefully) improve the overall quality of preclinical research. These impacts included 1) the influence of stakeholders at a higher level and changing the status quo, 2) increasing training for researchers, and 3) improving and investing in the education of the next generation of researchers.

Observing that their SR findings did not result in changes in their field, certain participants would have liked to pursue the issues at a higher level, for example by getting ethical committees and funders involved. It was suggested that preclinical SRs could become a requirement prior to performing an animal study and that this could be directed/checked by animal ethical committees. Funders could also be involved by emphasising the value of grant applications that include systematic overviews.

As noted above, questionnaire respondents were likely to recommend SRs to their colleagues and peers. This observation was confirmed in the interviews, with interviewees recommending that their peers or colleagues should receive proper training (via lectures, courses, and/or workshops) in the same way that they had. Participants made many suggestions about how to promote preclinical SRs in their field, including advertising on websites, in universities, on social media and at conferences, and by providing financial support. More investment in education would be beneficial in terms of promoting the value and acceptability of SRs, since they do not appear to be sufficiently understood nor employed in many fields.

Finally, participants reflected on the younger generation of scientists. Participants suggested that preclinical SRs and related training could be beneficial for new researchers.

> *"I think training is most powerful, because it's difficult to change the behaviour of the old researchers […]. But the young researchers you can definitely train, they can become knowledgeable in this field." – ZonMw72*

Discussion

Our findings have highlighted how conducting preclinical SRs impacted both research and researchers as a result of the MKMD "Knowledge infrastructure" module. Both questionnaire and interview data indicated that conducting preclinical SRs provided researchers with awareness, skills, and insights that

1) were used and promoted in the planning, conducting and reporting of their subsequent projects, 2) influenced their critical stance when appraising research, and 3) were used and promoted in their research fields (e.g., teaching, dissemination of results, advocating for more transparency). Ultimately, these impacts could lead to more transparent, high-quality research. To our knowledge, this is the first time qualitative research has been employed to identify the research impacts of preclinical SRs.

At a lab level, we saw that conducting preclinical SRs did not only provide insights and skills but also triggered long-lasting behavioural changes amongst our participants. Furthermore, most participants were willing to improve their work and to advocate for change in their teams (via support and teaching). We might extrapolate that if properly supported, coached, and empowered, a significant number of researchers might re-evaluate their research practices and follow the same path of reflection as our participants. This could lead to more teams wishing to improve knowledge transfer within their labs and among students, working as a type of SR feedback loop. Moreover, if universities invest in teaching about SRs and meta-analyses (e.g., on specific courses such as the legally required laboratory animal science courses for researchers wishing to perform animal studies (Function B course EU Directive 2010/63EU, Article 9 course in the Netherlands)), as suggested by our participants, this type of knowledge would become more universally accepted and might increase standards and expectations within the entire preclinical field.

However, knowledge transfer both within and between teams depends on several factors and antecedents, which can both promote and impede the dissemination of knowledge [36]. A legal framework and incentives from external stakeholders are needed to sustain this desire for improvement [29]. Given this context, incentives such as the MKMD scheme appear to provide appropriate support for initiating and sustaining improvements. Additionally, there are further external sources that could exert influence in this area. One of these are animal and human ethical committees, which could strengthen their requests by demanding SRs prior to researchers conducting new animal or human studies. Similar demands for transparency are happening with pre-registration

of preclinical studies, whose added value is increasing [37-39]. Other funding and regulatory bodies could further encourage the adoption of preclinical SRs by providing appropriate financial support or by promoting the value of preclinical SRs for translational purposes. Several organisations already support the use of preclinical SRs, such as the NC3Rs in the UK and NORECOPA [40, 41]. Lastly, journals could promote the adoption of preclinical SRs if more editors were familiar with their concept and value; to date, we found only four journals that regularly accept protocols for preclinical SRs [42-45].

With both legislation and intervention in place, only the pre-dispositions need to be set to trigger change, if we refer to the behaviour wheel of change framework. But, as highlighted in the interviews, several factors currently hinder the adoption and acceptance of preclinical SRs, including standard lab conventions, resistance from colleagues and supervisors, time pressure, lack of funding, competition, and lack of awareness of the value of preclinical SRs. There are international hurdles too, such as journals not yet willing to accept preclinical SRs and a lack of familiarity with SRs by journal editors and peer reviewers. Consequently, the emergence of preclinical SRs falls within a research culture paradox. On the one hand, high quality, transparent, and reproducible research is and should be the standard. Previous research highlighted that (preclinical) SRs can play a useful role in this context as they can provide helpful information and insights for policy decision making, and translation from preclinical to clinical studies [10, 46, 47]. On the other hand, researchers live in constant pursuit of delivering impactful results under great time pressure – leaving little space for conducting SRs, which are time-consuming exercise [48]. However, and as this case study illustrates, the time spent is beneficial in the sense that a change in mindset and behaviour is triggered and research quality is improved. Further studies are warranted to fully understand the barriers and facilitators to conduct preclinical SRs.

Regardless of all positive aspects, we should bear in mind that SRs rely on the studies they include and may therefore be of limited value. Like primary studies, they may also be constrained by poor reporting or poor conduct. Such limitations are highlighted with respect to clinical systematic reviews, for example with protocols in PROSPERO not corresponding to the PRISMA-P guidelines, and outcome

discrepancies between the protocol and final publication [49, 50]. Therefore, it is important that researchers continue to assess SR methodology and seek improvements [51-53]. On the preclinical side, Soliman et al., have provided new guidance for the appropriate conduct of SRs [54]. It is important to maintain a critical perspective and only use SRs where appropriate, with proper conduct and according to the proper guidelines.

Strengths and limitations of the study

The major strength of this study lies in its mixed methodology, using both questionnaires and interviews to identify research impacts. This triangulation of data increases the validity of our findings. The choice of design and methodology was carefully considered by researchers from different academic disciplines, helping to ensure the study's robustness.

Regardless of a relatively limited set target population, we collected sufficient data using both questionnaires and interviews. The impacts we identified mapped onto the Research Impact Framework for the categories: "type of knowledge/ problem", "research methods", "publication and papers", "translatability potential", "research network", and "communications", showing strong coherence between our findings and the Research Impact Framework. Our findings provide insight into how preclinical SRs have created impacts as a result of the ZonMw MKMD programme.

Despite significant strengths, we identified limitations in the methodology. First, our sample consisted mainly of Dutch researchers and was, of course, limited to researchers who were awarded a ZonMw grant within the knowledge infrastructure module. Consequently, our findings – enthusiasm and drive for improvements – may be related to the cultural context. The Netherlands are well known for their innovation-driven enterprise, so we cannot ensure that similar findings would emerge in a different context or with a foreign funding agency. Additionally, our findings are reported impacts, thus they are

subjective to the perceptions of the participants, in contrast to measurable, tangible impacts. Nevertheless, to our knowledge, this is the first case study on such a topic and in the future, when preclinical SRs become more established, it would be interesting to repeat this study on a broader scale, for example within European institutes and/or the Ensuring Value in Research Funder Forum.

Second, because this study had to be conducted over the summer, it is possible that, despite our best efforts to obtain a high response rate, the summer holidays reduced the availability of participants. Furthermore, the time scale for this study was rather short (3 months), which put pressure on the time we could spend on collecting and analysing our data. Finally, data collection, coding and analysis of the transcripts were all performed by one researcher.

Conclusion

This case study has provided important insights into the impacts of training, coaching and conducting preclinical SRs on both research and researchers. Not only did SRs impact research at a lab level, they led to changes in researchers' views and critical abilities, and spurred efforts to promote change for the better within their fields. Our project highlights the necessity and importance of supporting preclinical researchers to perform SRs, and demonstrates the impact of this on the quality and transparency of research, as well as on researchers' awareness and motivation to change the status quo. Our findings suggest that similar support, as provided here by the Dutch funding agency ZonMw, is relevant and should be encouraged internationally, contributing to the improved quality and translation of preclinical research

Acknowledgement

We would like to thank Janne Swinkels, Stevie van der Mierden, Franck Meijboom, Brett Lidbury and Milan Khanpour, for piloting and providing feedback on questionnaire and/or interview guide. As well, we thank Rob de Vries for his support in triangulation and his feedback on the questionnaire and interview guide.

References

1. STM: International Association of Scientific TaMP. The STM Report : An overview of scientific and scholarly publishing Prins Willem Alexanderhof 5, The Hague, 2595BE, The Netherlands International Association of Scientific, Technical and Medical Publishers 2018.
2. AJE Scholarly Publishing Report: 2016 American Journal Experts 2016.
3. Cochrane Handbook for Systematic Reviews of Interventions Version 5.1.0 [updated March 2011]: The Cochrane Collaboration; 2011 01/02/2021]. Available from: www.handbook.cochrane.org.
4. Chalmers I, Hedges LV, Cooper H. A brief history of research synthesis. Eval Health Prof. 2002;25(1):12-37, doi:10.1177/0163278702025001003
5. Hong QN, Pluye P. Systematic reviews: A brief historical overview. Education for Information. 2018;34:261-76, doi:10.3233/EFI-180219
6. Page MJ, Shamseer L, Tricco AC. Registration of systematic reviews in PROSPERO: 30,000 records and counting. Syst Rev. 2018;7(1):32, doi:10.1186/s13643-018-0699-4
7. van Luijk J, Bakker B, Rovers MM, Ritskes-Hoitinga M, de Vries RB, Leenaars M. Systematic reviews of animal studies; missing link in translational research? PLoS One. 2014;9(3):e89981, doi:10.1371/journal.pone.0089981

8. Horn J, de Haan RJ, Vermeulen M, Luiten PG, Limburg M. Nimodipine in animal model experiments of focal cerebral ischemia: a systematic review. Stroke. 2001;32(10):2433-8, doi:10.1161/hs1001.096009

9. Pound P, Ritskes-Hoitinga M. Can prospective systematic reviews of animal studies improve clinical translation? J Transl Med. 2020;18(1):15, doi:10.1186/s12967-019-02205-x

10. Ritskes-Hoitinga M, Leenaars M, Avey M, Rovers M, Scholten R. Systematic reviews of preclinical animal studies can make significant contributions to health care and more transparent translational medicine. Cochrane Database Syst Rev. 2014(3):ED000078, doi:10.1002/14651858.ED000078

11. CAMARADE's website: University of Edinburgh; 2020 [01/02/2021]. Available from: https://www.ed.ac.uk/clinical-brain-sciences/research/camarades/about-camarades.

12. Syrcle's website 2020 [01/02/2021]. Available from: www.syrcle.nl.

13. National Institute for Health Research: Registering a review of animal studies on PROSPERO 2020 [Available from: http://cdn.elsevier.com/promis_misc/PROSPEROAnimal.pdf.

14. SABRE Research UK 2019 [01/02/2021]. Available from: https://en.wikipedia.org/wiki/SABRE_Research_UK.

15. ZonMw's website - Programme "More knowledge with fewer animals" 2020 [01/02/2021]. Available from: https://www.zonmw.nl/en/research-and-results/fundamental-research/programmas/programme-detail/more-knowledge-with-fewer-animals/.

16. ZonMw's website - Modules - Programme "More knowledge with fewer animals" 2020 [01/02/2021]. Available from: https://www.zonmw.nl/en/programma-opslag-en/more-knowledge-with-fewer-animals/modules/.

17. Research Impact Principles and Framework - Glossary Australia: Australian Research Council; 2019 [cited 2020 05/02/2021]. Available from: https://www.arc.gov.au/policies-strategies/strategy/research-impact-principles-framework.

18. Adam P, Ovseiko PV, Grant J, Graham KEA, Boukhris OF, Dowd AM, et al. ISRIA statement: ten-point guidelines for an effective process of research impact assessment. Health Res Policy Syst. 2018;16(1):8, doi:10.1186/s12961-018-0281-5

19. Measuring research: A guide to research evaluation frameworks and tools. Cambridge: Association of American Medical Colleges; 2013.

20. Rapple C. Research impact: what it is, why it matters, and how you can increase impact potential 2019 [07/2020]. Available from: https://blog.growkudos.com/research-mobilization/research-impact-what-why-how#:~:text=Impact%20is%20important%20because%20it,who%20may%20benefit%20from%20it.

21. Research impact assessment plan - Guidelines Version 2.0. A guide for completing the Research Impact Assessment (RIA) Plan The International School on Research Impact Assessment 2014.

22. Fryirs KA, Brierley GJ, Dixon T. Engaging with research impact assessment for an environmental science case study. Nat Commun. 2019;10(1):4542, doi:10.1038/s41467-019-12020-z

23. Graham KER, Langlois-Klassen D, Adam SAM, Chan L, Chorzempa HL. Assessing Health Research and Innovation Impact: Evolution of a Framework and Tools in Alberta, Canada. Frontiers in Research Metrics and Analytics. 2018;3doi:10.3389/frma.2018.00025

24. Watermeyer R. Issues in the articulation of ‘impact’:the responses of UK academics to ‘impact’ as a new measure of research assessment. Studies in Higher Education. 2014;39(2):359-77, doi:10.1080/03075079.2012.709490

25. Greenhalgh T, Raftery J, Hanney S, Glover M. Research impact: a narrative review. BMC Med. 2016;14:78, doi:10.1186/s12916-016-0620-8

26. Excellence in Research for Australia: Australian Research Council; 2019 [05/02/2021]. Available from: https://www.arc.gov.au/excellence-research-australia.

27. Making an Impact: A Preferred Framework and Indicators to Measure Returns on Investment in Health Research. Ottawa, ON, Canada: Canadian Academy of Health Sciences; 2009.

28. Kuruvilla S, Mays N, Pleasant A, Walt G. Describing the impact of health research: a Research Impact Framework. BMC Health Serv Res. 2006;6:134, doi:10.1186/1472-6963-6-134

29. Michie S, van Stralen MM, West R. The behaviour change wheel: a new method for characterising and designing behaviour change interventions. Implement Sci. 2011;6:42, doi:10.1186/1748-5908-6-42

30. West R, Michie S. A brief introduction to the COM-B Model of behaviour and the PRIME Theory of motivation. Qeios. 2020doi:10.32388/WW04E6.2

31. Tong A, Sainsbury P, Craig J. Consolidated criteria for reporting qualitative research (COREQ): a 32-item checklist for interviews and focus groups. Int J Qual Health Care. 2007;19(6):349-57, doi:10.1093/intqhc/mzm042

32. O'Brien BC, Harris IB, Beckman TJ, Reed DA, Cook DA. Standards for reporting qualitative research: a synthesis of recommendations. Acad Med. 2014;89(9):1245-51, doi:10.1097/ACM.0000000000000388

33. Finstad K. Response Interpolation and Scale Sensitivity: Evidence Against 5-Point Scales. Journal of usability studies. 2010;5(3):104-10,

34. Maguire M, Delahunt B. Doing a Thematic Analysis: A Practical, Step-by-Step Guide for Learning and Teaching Scholars. AISHE-J. 2017;8(3)

35. Aluwihare-Samaranayake D. Ethics in Qualitative Research: A View of the Participants' and Researchers' World from a Critical Standpoint. International Journal of Qualitative Methods. 2012;11(2):64-81, doi:10.1177/160940691201100208

36. Shahbaznezhad H, Rashidirad M, Vaghefi I. A systematic review of the antecedents of knowledge transfer: an actant-object view. European Business Review. 2019;31(6):970-95, doi:10.1108/EBR-07-2018-0133

37. Bert B, Heinl C, Chmielewska J, Schwarz F, Grune B, Hensel A, et al. Refining animal research: The Animal Study Registry. PLoS Biol. 2019;17(10):e3000463-e, doi:10.1371/journal.pbio.3000463

38. Preclinical trials.eu website [09/02/2021]. Available from: https://preclinicaltrials.eu/.

39. van der Naald M, Wenker S, Doevendans PA, Wever KE, Chamuleau SAJ. Publication rate in preclinical research: a plea for preregistration. BMJ Open Science. 2020;4(1):e100051, doi:10.1136/bmjos-2019-100051

40. NC3R website [09/02/2021]. Available from: https://www.nc3rs.org.uk/camaradesnc3rs-systematic-review-facility-syrf.

41. NORECOPA website [09/02/2021]. Available from: https://norecopa.no/more-resources/literature-searches-and-systematic-reviews.

42. BMJ open website: BMJ Publishing Group Ltd.; [09/02/2021]. Available from: https://bmjopen.bmj.com/.

43. Systematic Reviews website: BioMed Central Ltd [09/02/2021]. Available from: https://systematicreviewsjournal.biomedcentral.com/.

44. Environment International website: Elsevier B.V.; [09/02/2021]. Available from: https://www.journals.elsevier.com/environment-international/editors-choice/focus-on-systematic-reviews-and-systematic-evidence-maps.

45. Laboratory Animals website [09/02/2021]. Available from: https://journals.sagepub.com/home/lan.

46. Bunn F, Trivedi D, Alderson P, Hamilton L, Martin A, Iliffe S. The impact of Cochrane Systematic Reviews: a mixed method evaluation of outputs from Cochrane Review Groups supported by the UK National Institute for Health Research. Systematic Reviews. 2014;3(1):125, doi:10.1186/2046-4053-3-125

47. Hooijmans CR, de Vries RBM, Ritskes-Hoitinga M, Rovers MM, Leeflang MM, IntHout J, et al. Facilitating healthcare decisions by assessing the certainty in the evidence from preclinical animal studies. PLoS One. 2018;13(1):e0187271, doi:10.1371/journal.pone.0187271

48. Borah R, Brown AW, Capers PL, Kaiser KA. Analysis of the time and workers needed to conduct systematic reviews of medical interventions using data from the PROSPERO registry. BMJ Open. 2017;7(2):e012545, doi:10.1136/bmjopen-2016-012545

49. Booth A, Mitchell AS, Mott A, James S, Cockayne S, Gascoyne S, et al. An assessment of the extent to which the contents of PROSPERO records meet the systematic review protocol reporting items in PRISMA-P. F1000Research. 2020;9:773, doi:10.12688/f1000research.25181.1

50. Tricco AC, Cogo E, Page MJ, Polisena J, Booth A, Dwan K, et al. A third of systematic reviews changed or did not specify the primary outcome: a PROSPERO register study. Journal of clinical epidemiology. 2016;79:46-54, doi:10.1016/j.jclinepi.2016.03.025

51. Kahale LA, Diab B, Brignardello-Petersen R, Agarwal A, Mustafa RA, Kwong J, et al. Systematic reviews do not adequately report or address missing outcome data in their analyses: a methodological survey. Journal of clinical epidemiology. 2018;99:14-23, doi:10.1016/j.jclinepi.2018.02.016

52. Robson RC, Pham B, Hwee J, Thomas SM, Rios P, Page MJ, et al. Few studies exist examining methods for selecting studies, abstracting data, and appraising quality in a systematic review. Journal of clinical epidemiology. 2019;106:121-35, doi:10.1016/j.jclinepi.2018.10.003

53. Waffenschmidt S, Knelangen M, Sieben W, Bühn S, Pieper D. Single screening versus conventional double screening for study selection in systematic reviews: a methodological systematic review. BMC medical research methodology. 2019;19(1):132, doi:10.1186/s12874-019-0782-0

54. Soliman N, Rice ASC, Vollert J. A practical guide to preclinical systematic review and meta-analysis. PAIN. 2020;161(9)

Supporting information

S1 Appendix: The Research Impact Framework

Table 1: Research Impact Framework – first main area of impact: research-related impacts (modified from Kuruvilla et al., 2007)

S2 Appendix: The Behaviour Change Wheel

Figure 2: The Behaviour Change Wheel (modified from Michie et al., 2011)

S3 Appendix: Standards for Reporting Qualitative Research (SQRQ) checklist

S4 Appendix: Consolidated criteria for reporting qualitative studies (COREQ): 32-item checklist

S5 Appendix: Invitation to complete the online questionnaire and reminder

S6 Appendix: Full questionnaire

S7 Appendix: Informed consent form

S8 Appendix: Interview guide

S9 Appendix: Code tree and harmonisation

S10 Appendix Organisation of the questionnaire and number of respondents per questions

S11 Appendix: Questionnaire results for questions on planning, designing and reporting animal experiments, and appraising research after conducting a preclinical SR

Figure 11.1: Overview of answers on planning and designing animal experiments after conducting a preclinical SR

Figure 11.2: Overview of answers on writing about animal experiments after conducting a preclinical SR

Figure 11.3 Overview of answers on appraising research

S12 Appendix: Skills learnt and improved by performing preclinical systematic review

Table 12: Overview of skills learnt and improved by participants after conducting (part of) their preclinical SR

S13 Appendix: Questionnaire results

Figure 1 Click here to access/download;Figure;Fig1.tif

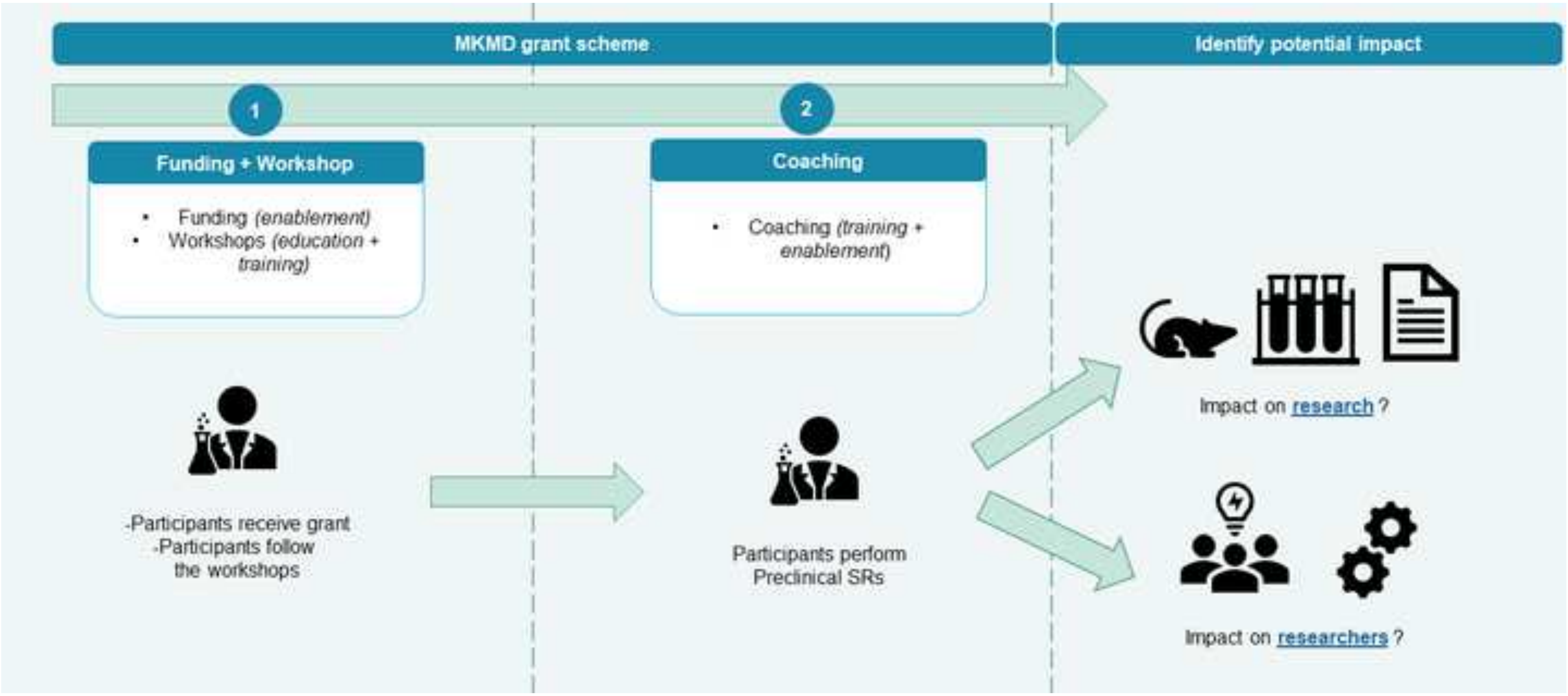

 Click here to access/download;Figure;Fig2.tif

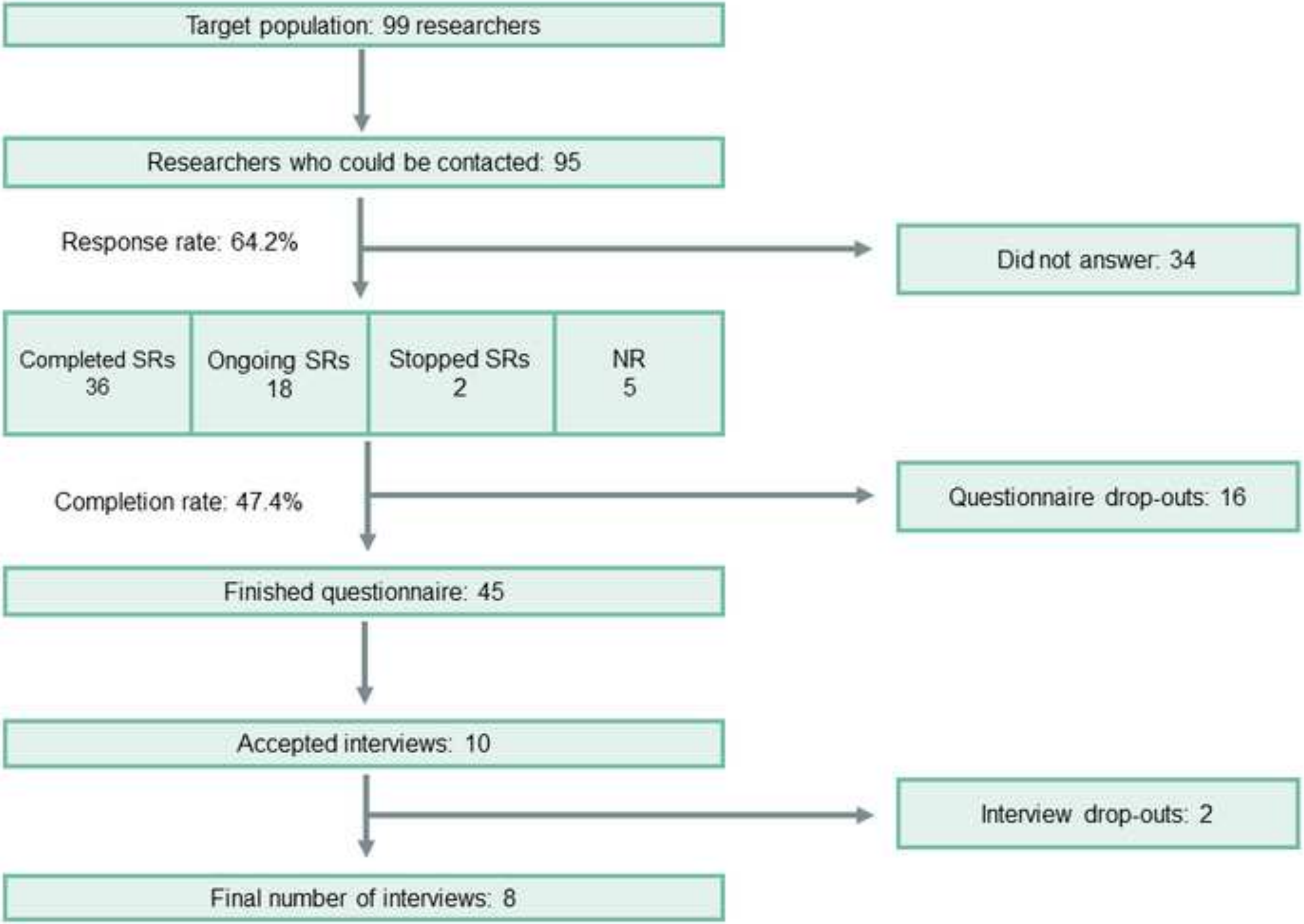

Figure 3A

Click here to access/download;Figure;Fig3A.tif

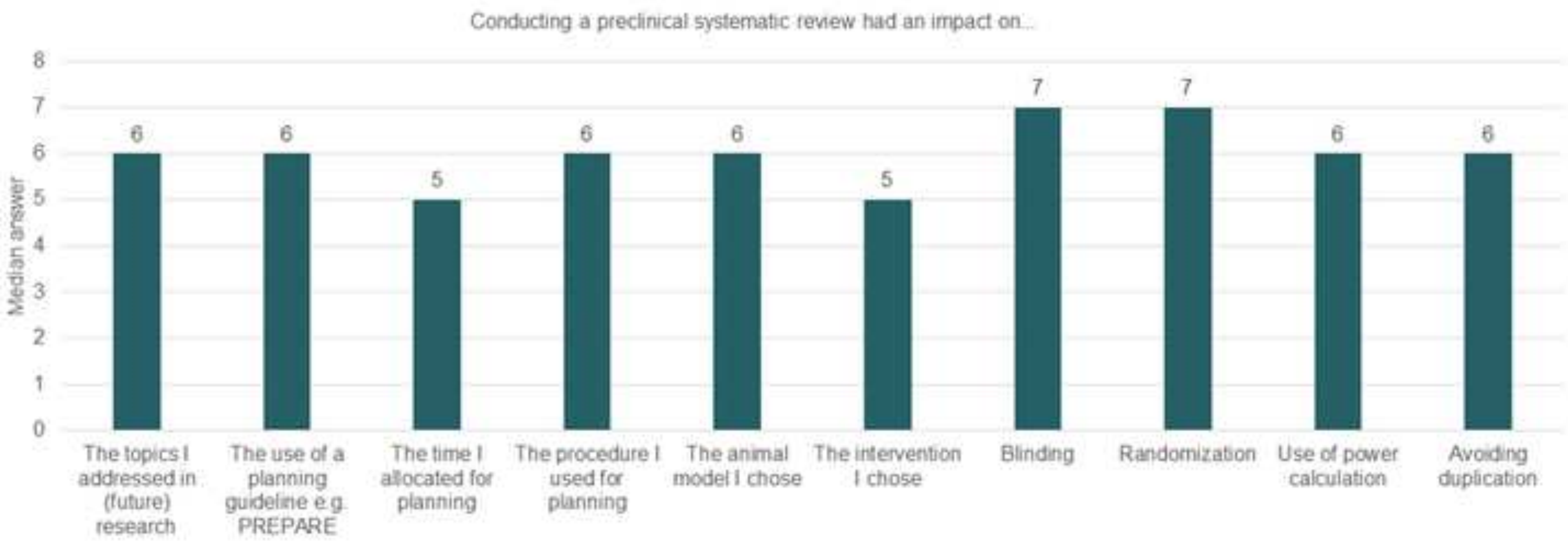

Figure 3B

Click here to access/download;Figure;Fig3B.tif

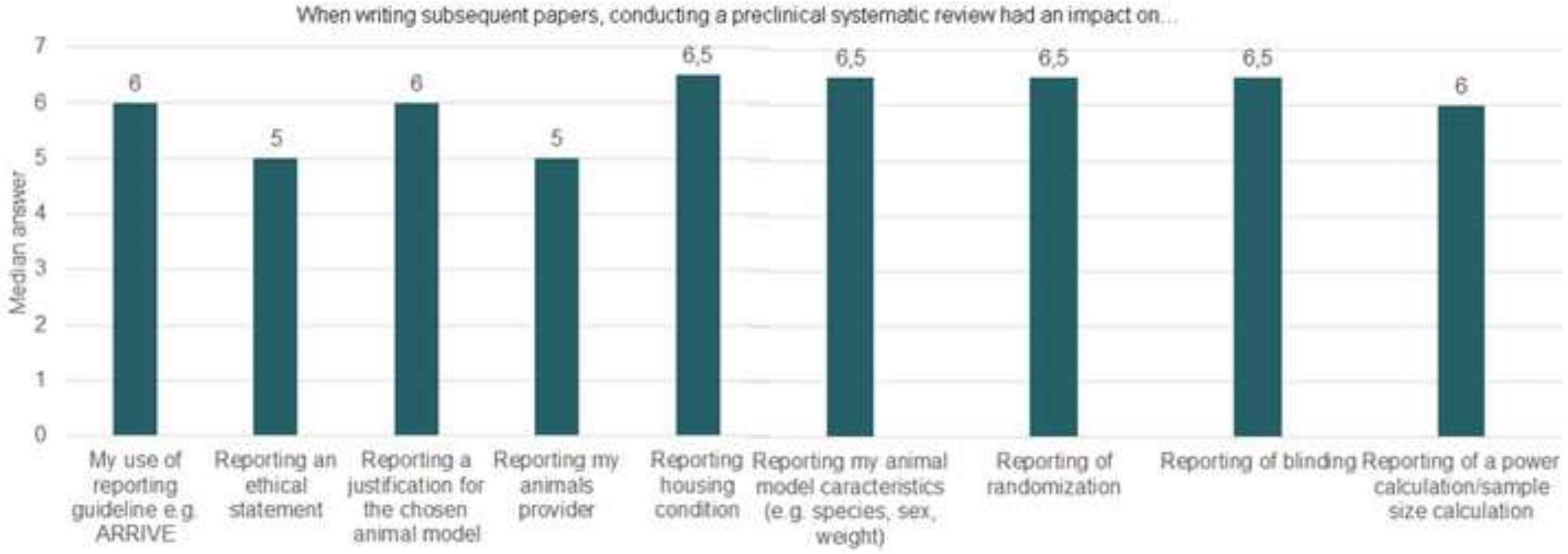

Figure 4

Click here to access/download;Figure;Fig4.tif

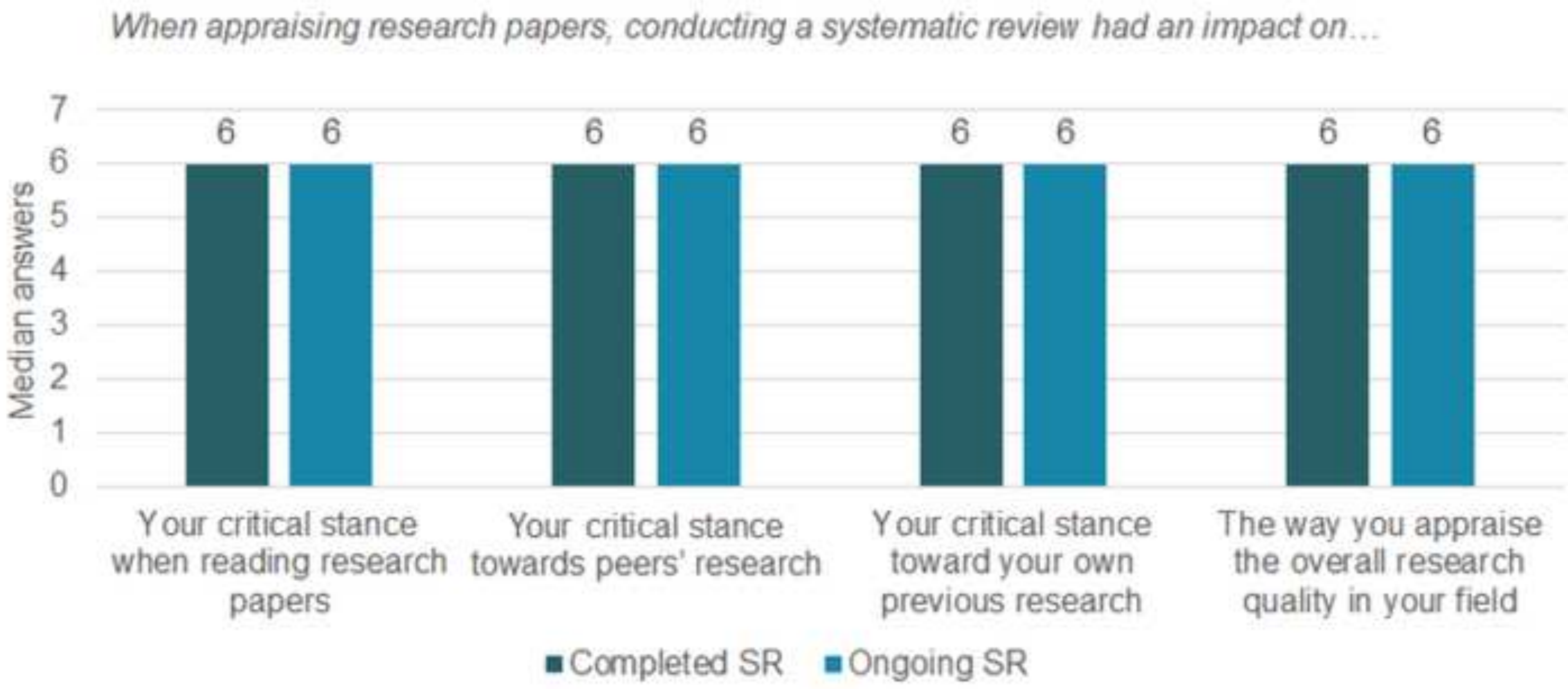

Figure 5

Click here to access/download;Figure;Fig5.tif

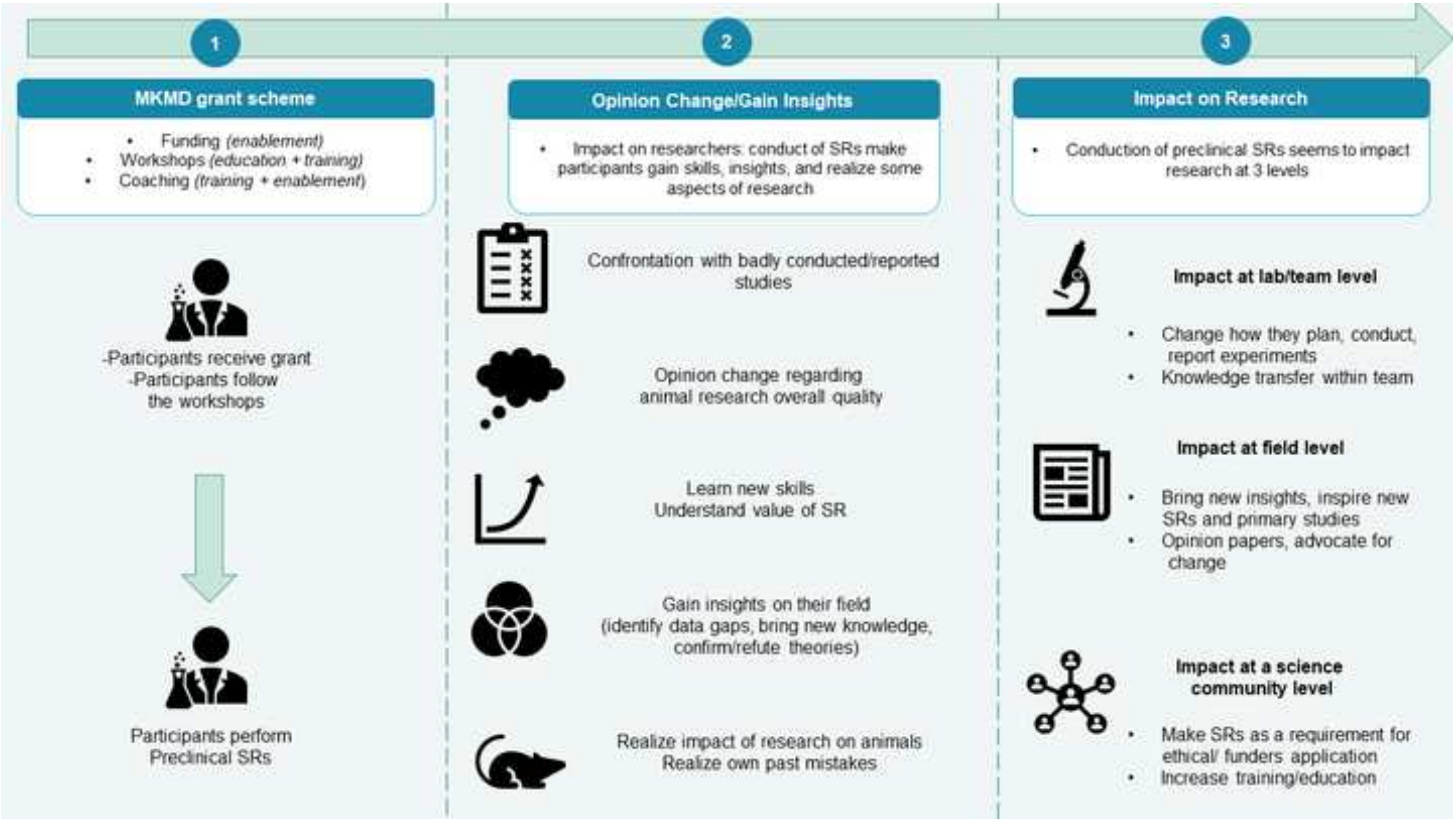

Click here to access/download
Supporting Information
S1 Appendix_Table 1.docx

Click here to access/download
Supporting Information
S2 Appendix_Fig2.docx

Click here to access/download
Supporting Information
S3 Appendix.docx

S4 Appendix

Click here to access/download
Supporting Information
S4 Appendix.docx

Click here to access/download
Supporting Information
S5 Appendix.docx

Click here to access/download
Supporting Information
S6 Appendix.docx

S7 Appendix

Click here to access/download
Supporting Information
S7 Appendix.docx

S8 Appendix

Click here to access/download
Supporting Information
S8 Appendix.docx

Click here to access/download
Supporting Information
S9 Appendix.docx

Click here to access/download
Supporting Information
S10 Appendix.docx

Click here to access/download
Supporting Information
S11 Appendix_Fig11.1_Fig11.2_Fig11.3.docx

Click here to access/download
Supporting Information
S12 Appendix_Table12.docx

Click here to access/download
Supporting Information
S13 Appendix.pdf
