# Supplementary material for: The impact of conducting preclinical systematic reviews on researchers and their research: A mixed method case study
Source: PLoS One. 2021 Dec 13;16(12):e0260619. doi: 10.1371/journal.pone.0260619 (PMC8668092; doi:10.1371/journal.pone.0260619)
Supplement: Attachment — Submitted filename: Reponse to reviewers.docx [file pone.0260619.s016.docx]

Dear Editors,

We would like to express to both editors and reviewers our sincere thank you for their comments and the opportunity to revise our submitted manuscript.

We have addressed the remaining issues that were raised by reviewer 2 below.

We genuinely hope that our revisions will be satisfactory and that our manuscript will be now suitable for publication.

Thank you again for considering our manuscript for publication in PLoS One.

Looking forward to hearing from you again,

Sincerely,

On behalf of all co-authors,

Julia Menon

**Reviewer’s comments**

1. Line 249: I think '(associate)' can be removed from the text entirely, i.e. "...namely research assistants (n=2), a PhD student (n=1) and professors (n=4)."

Authors’ response: *We agree that this term makes the sentence “wordy” and does not per se add to the context. We have removed it as suggested.*

2. Line 523: I take your point on the intention behind "...had not (yet) made..." in the previous version, and that this reflects the wishes of the participant rather than you as an author team. While I do think it's better to steer clear from any wording that could result in perceived bias, you could consider changing the sentence to "...discovering that the insights from their SRs appeared to have had no impact on their field to date." This is entirely up to you, as the revised version you provided is also fine, but perhaps this provides a bit more nuance.

Authors’ response: *Thank you for this suggestion! We feel it indeed brings more nuance and clarity to this part of the text. The sentence was modified as suggested.*
